# Cilia-localized GID/CTLH ubiquitin ligase complex regulates protein homeostasis of sonic hedgehog signaling components

**DOI:** 10.1242/jcs.259209

**Published:** 2022-05-11

**Authors:** Friederike Hantel, Huaize Liu, Lisa Fechtner, Herbert Neuhaus, Jie Ding, Danilo Arlt, Peter Walentek, Pablo Villavicencio-Lorini, Christoph Gerhardt, Thomas Hollemann, Thorsten Pfirrmann

**Affiliations:** 1 Institute of Physiological Chemistry, Martin-Luther University Halle-Wittenberg, 06114 Halle, Germany; 2 Renal Division, Department of Medicine, University Hospital Freiburg, Freiburg University Faculty of Medicine, 79106 Freiburg, Germany; 3 CIBSS – Centre for Integrative Biological Signalling Studies, University of Freiburg, 79104 Freiburg, Germany; 4 Institute of Human Genetics, Martin-Luther University Halle-Wittenberg, 06112 Halle, Germany; 5 Department of Medicine, Health and Medical University, 14471 Potsdam, Germany

**Keywords:** AMPK, Ubiquitin, Cilium, Ciliopathies, Sonic hedgehog, GID complex

## Abstract

Cilia are evolutionarily conserved organelles that orchestrate a variety of signal transduction pathways, such as sonic hedgehog (SHH) signaling, during embryonic development. Our recent studies have shown that loss of GID ubiquitin ligase function results in aberrant AMP-activated protein kinase (AMPK) activation and elongated primary cilia, which suggests a functional connection to cilia. Here, we reveal that the GID complex is an integral part of the cilium required for primary cilia-dependent signal transduction and the maintenance of ciliary protein homeostasis. We show that GID complex subunits localize to cilia in both *Xenopus laevis* and NIH3T3 cells. Furthermore, we report SHH signaling pathway defects that are independent of AMPK and mechanistic target of rapamycin (MTOR) activation. Despite correct localization of SHH signaling components at the primary cilium and functional GLI3 processing, we find a prominent reduction of some SHH signaling components in the cilium and a significant decrease in SHH target gene expression. Since our data reveal a critical function of the GID complex at the primary cilium, and because suppression of GID function in *X. laevis* results in ciliopathy-like phenotypes, we suggest that GID subunits are candidate genes for human ciliopathies that coincide with defects in SHH signal transduction.

## Abstract (precis)

**Summary:** The GID ubiquitin ligase complex localizes to primary cilia, influences sonic hedgehog signaling and causes phenotypes reminescent of ciliopathies.

## INTRODUCTION

**Fig. 1.**
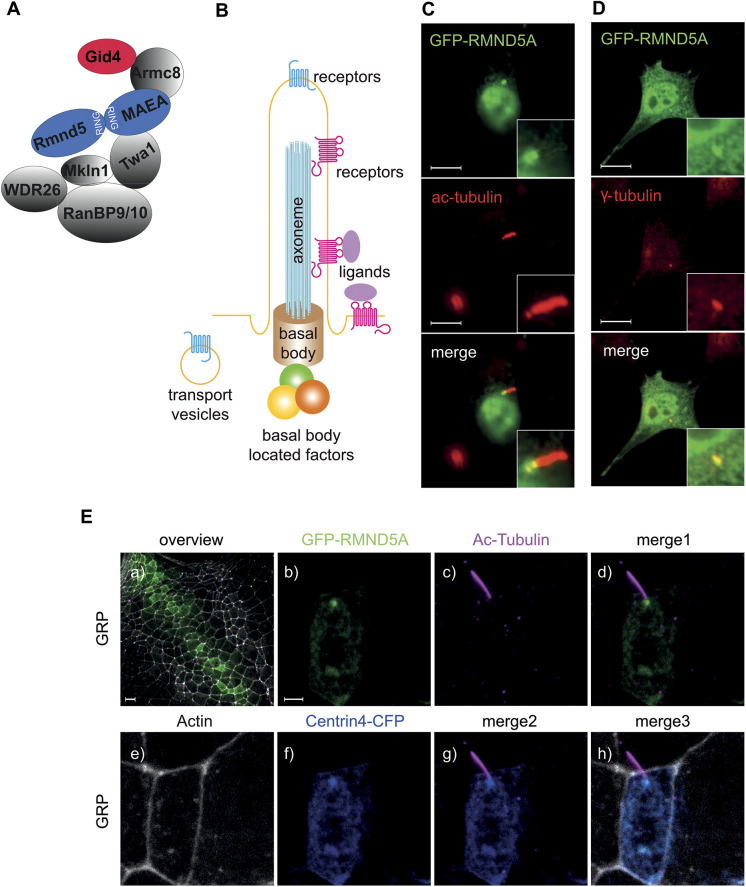
**GID subunits colocalize to the basal body of mono-ciliated cells.** (A) Schematic model of the vertebrate GID complex and its known subunits; RING domain-containing subunits are highlighted in blue, the substrate-recruiting factor GID4 is highlighted in red. (B) Schematic model of the primary cilium, composed of a basal body, axoneme and ciliary membrane. A large number of transporters, structural proteins, membrane receptors and basal body-located factors function in the primary cilium. (C,D) RMND5A localizes to the basal body of the primary cilium in NIH-3T3 cells. Cells were transfected with plasmids encoding GFP–RMND5A for 24 h and then further serum-starved (high glucose DMEM, 0.5% FCS) for an additional 24 h to induce ciliogenesis. After fixation, cells were stained for (C) acetylated tubulin (ac-tubulin) or (D) γ-tubulin to visualize the axoneme or the basal body of the primary cilium, respectively. Images were merged to identify overlapping signals (merge, yellow). Inset images show the magnification of a primary cilium and the basal body. Images shown in C and D are representative of seven and four images, respectively. Scale bars: 10 μm. (E) Rmnd5a localizes to basal bodies of motile mono-cilia of the GRP in *X. laevis*. mRNAs encoding GFP–Rmnd5a (green) and Centrin4–CFP (blue, centrioles/basal bodies) were injected at the four-cell stage. Embryos were fixed and stained at NF stage 17 to visualize cilia (magenta, ac-tubulin) and actin (white). Merge 1 shows a merged image of Eb and Ec; merge 2 shows a merged image of Ec and Ef; and merge 3 shows a merged image of Ee, Ef and Ec. Images are *z*-projections of confocal micrographs and represent five biological samples derived from one experiment. Scale bars: 20 μm (Ea), 3 μm (Eb–h).

Cilia are hair-like structures that are present on almost every vertebrate cell. At the core of a cilium is a microtubule-based axoneme, which extends from the basal body and contains, in cross section, a ring of nine duplet microtubules surrounding a central duplet (Fig. 1B) (Wheatley, 1995; Wheatley et al., 1996). The ciliary membrane is a discrete compartment and contains various receptor proteins, allowing primary cilia to act as signaling hubs that regulate, among others, cell proliferation, cell differentiation and polarity (Berbari et al., 2009; Goetz and Anderson, 2010; Reiter and Leroux, 2017). Primary cilia also function as cellular antennae that sense and react to nutritional, chemical and mechanical stimuli (Boehlke et al., 2010; Song et al., 2018). Consequently, dysfunction of primary cilia manifests in different hereditary organ-specific or syndromic diseases, collectively known as ciliopathies (Ansley et al., 2003; Fliegauf et al., 2007; Hildebrandt et al., 2011; Pazour et al., 2002; Reiter and Leroux, 2017).

Among the many pathways that require primary cilia, the sonic hedgehog (SHH) signaling pathway is the most intensively studied (Carballo et al., 2018; Patel et al., 2017; Sasai et al., 2019; Teperino et al., 2014). This pathway is well known for its role in patterning multiple regions of the developing embryo. In particular, it affects neural crest cell survival and craniofacial development (Doro et al., 2019; Liu, 2016; Szabo-Rogers et al., 2010), development of the visual nervous system (Cavodeassi et al., 2019), cell differentiation of the eye as well as dorsoventral patterning of the nervous system (Andrews et al., 2019; Pal and Mukhopadhyay, 2015). A subset of developmental phenotypes typical for syndromic ciliopathies are due to simultaneously altered SHH signaling (Asadollahi et al., 2018; Doherty, 2009; Lee and Gleeson, 2011). Protein homeostasis in the cilium is crucial for proper SHH signaling and is achieved by regulated entry, exit and degradation of proteins at the ciliary base (Malicki and Avidor-Reiss, 2014). The transition zone serves as a diffusion barrier at the ciliary base and is crucial for the transition of proteins into and out of the cilium (Jensen and Leroux, 2017). Protein complexes like the BBSome complex (associated with Bardet–Biedl syndrome), the MKS complex (associated with Meckel–Gruber syndrome), and the NPHP complex (associated with nephronophthisis), as well as Septin-2, are critical for this process (Long and Huang, 2019). Aberrations in SHH signaling, in particular mutations affecting PTCH1 receptor, are also implicated in the initiation and progression of multiple types of cancers, including medulloblastoma (Rudin et al., 2009), breast cancer (Kasper et al., 2009), basal cell carcinoma (Yu et al., 2014), Burkitt lymphoma (Yoon et al., 2013) and pancreatic cancer (Lauth and Toftgård, 2011). A role for the primary cilium in the regulation of cellular energy homeostasis via AMP-activated protein kinase (AMPK) has also been described recently (Boehlke et al., 2010; Orhon et al., 2016; Pampliega et al., 2013). Defects in these processes are likely a cause of type 2 diabetes and morbid obesity, often accompanied by ciliopathies such as Bardet–Biedl syndrome (BBS) and Almström syndrome (Tsang et al., 2018a,b). Interestingly, the SHH pathway affects autophagy via AMPK (Xiao et al., 2015; Xu et al., 2014), whereas AMPK, in turn, inhibits SHH signaling in several human cell lines and directly phosphorylates and destabilizes the SHH-dependent transcription factor GLI1 (Di Magno et al., 2016; Li et al., 2015).

The GID/CTLH protein complex (referred to herein as the GID complex) belongs to an evolutionarily conserved family of multi-subunit E3 ubiquitin ligases (Francis et al., 2013). In *Saccharomyces cerevisiae*, the GID complex regulates the metabolic switch from gluconeogenesis to glycolysis by targeting key enzymes of gluconeogenesis for 26S proteasomal degradation (Chen et al., 2017; Liu and Pfirrmann, 2019; Santt et al., 2008). Recently, the structure of the yeast GID complex has been solved (Qiao et al., 2019) and the function of Gid4 (also known as Vid24) in substrate recognition has been described in molecular detail (Chen et al., 2017). Additionally, a new GID subunit with similarities to Gid4, named Gid10, has been found to function as a substrate recognition factor (Melnykov et al., 2019). The individual subunits of the yeast GID complex, Gid1 (also known as Vid30), Gid2 (Rmd5), Gid4, Gid5 (Vid28), Gid7, Gid8 and Gid9 (Fyv10), find their human orthologs in RANBP9 and RANBP10 (Ran-binding proteins 9 and 10), RMND5A and RMND5B (required for meiotic nuclear division 5 homologs A and B), GID4 (GID complex subunit 4 homolog), ARMC8 (armadillo repeat-containing protein 8), MKLN1 (muskelin 1) or WDR26 (WD repeat-containing protein 26), TWA1 (also known as GID8, GID complex subunit 8 homolog), and MAEA (macrophage erythroblast attacher), respectively. These GID subunits are components of the human GID complex (Fig. 1A; Table S1) (Texier et al., 2014). Functions of the vertebrate GID complex include cell proliferation (Lampert et al., 2018), regulation of cell metabolism (Leal-Esteban et al., 2018), regulation of AMPK (Liu et al., 2019), and erythropoiesis (Zhen et al., 2019). The subunits MAEA and RMND5A contain non-canonical RING domains that are crucial for ubiquitin ligase activity (Liu and Pfirrmann, 2019; Pfirrmann et al., 2015; Santt et al., 2008). A recent systems biology approach to define a primary cilium protein–protein interaction network has suggested a function of the GID complex in primary cilia (Boldt et al., 2016).

Here, we show that the GID ubiquitin ligase complex has a fundamental function at the cilium and is required for primary cilia-dependent SHH signal transduction. Subunits of the GID complex localize at the basal body of primary cilia and motile monocilia, and we found that SHH signaling is severely disturbed in cells lacking GID complex function. Interestingly, ciliary proteins, including the SHH components suppressor of fused homolog (SUFU), PTCH1 and GLI2, still localize to the primary cilium, and GLI3 processing is fully functional. However, we find reduced concentrations of different SHH components in the primary cilium but not the cytosol. This suggests a function of the cilia-localized GID complex in regulating proteostasis of several SHH signaling components within the primary cilium. Pharmacologically induced changes in the activity of the AMPK–mechanistic target of rapamycin (MTOR) signaling axis interfere with SHH signaling independently of the GID complex, suggesting that substrates other than AMPK are targeted by the GID complex. The distinct function of the GID complex at the primary cilium is further substantiated in our model organism *Xenopus laevis*, where we find subunits of the GID complex expressed in organs that require cilia for proper development. Depletion of GID complex subunits results in several ciliopathy-like developmental phenotypes reminiscent of the more severe spectrum. Overall, we propose that the GID complex, as an integral part of cilia, is critical for the maintenance of protein homeostasis for several SHH components, and we hypothesize that genes encoding GID complex subunits are novel disease gene candidates for human ciliopathies.

## RESULTS

### GID complex subunits colocalize with primary cilia and centrioles

**Fig. 2.**
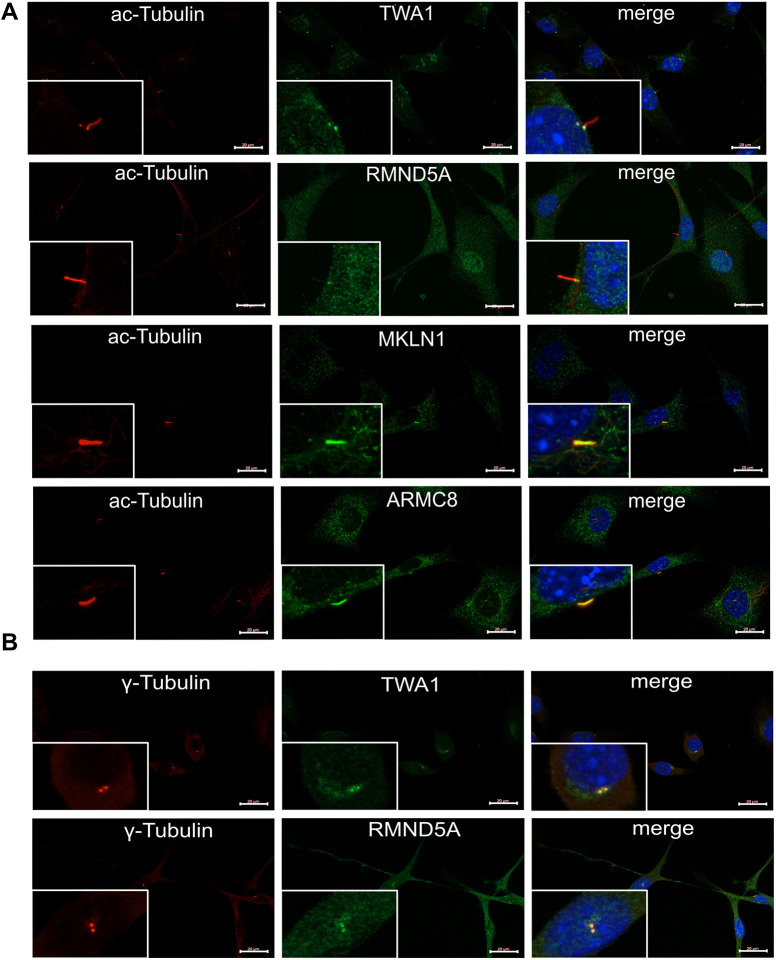
**GID complex subunits colocalize with primary cilia and centrioles.** (A) Cells were serum-starved (high glucose DMEM, 0.5% FCS) for 24 h to induce ciliogenesis. After fixation, cells were stained for acetylated tubulin (ac-tubulin) to visualize the axoneme of the primary cilium. Additional antibodies were diluted 1:100 in PBS containing 3% BSA and 0.3% Tween-20 and used to visualize subunits of the GID complex (TWA1, RMND5A, MKLN1 and ARMC8). Nuclei were stained using DAPI (blue). (B) As described in A without prior serum starvation and stained for γ-tubulin to visualize the basal body of the primary cilium. Images in A and B were merged to identify overlapping signals (merge, yellow). Inset images show a cell with a primary cilium at original size at the respective magnifications. Scale bars: 20 μm. Percentage of cells with ac-Tubulin colocalization: TWA1, 75.9%, *n*=29; RMND5A, 60.7%, *n*=28; ARMC8, 96.3%, *n*=27; MKLN1, 93.1%, *n*=29. Percentage of cells with γ-tubulin colocalization: TWA1, 87.1%, *n*=31; RMND5A, 82.8%, *n*=29; *n*=total number of cells investigated.

A previous proteomics study has identified the GID complex at the basal body (Boldt et al., 2016). We therefore investigated whether the GID complex is a functional unit of the cilium using *in vitro* and *in vivo* approaches. Previously, we have monitored RMND5A localization under standard growth conditions and found that it is most prominent in the nucleus and cytosol (Pfirrmann et al., 2013). To test for colocalization with primary cilia, we transfected NIH-3T3 cells using plasmids encoding a GFP–RMND5A fusion protein and arrested cells in G0 by serum starvation. Under these conditions, most cells contained a primary cilium that could be visualized using an antibody against acetylated tubulin, which is enriched in the axoneme (Fig. 1C,E). Indeed, a clear GFP–RMND5A signal appeared proximal to the axoneme with a defined partial overlap, most likely at the basal body, which was labeled with anti-γ-tubulin antibodies (Fig. 1D). The presence of RMND5A in primary cilia prompted us to also look at other types of cilia. Motile mono-ciliated cells are present in the epithelium of the gastrocoel roof plate (GRP) in the developing *X. laevis* tadpole (Walentek and Quigley, 2017). In these epithelial cells, we observed Rmnd5a (also known as Gid2 in *X. laevis*) proximal to the axoneme of motile cilia (Fig. 1Ec) with a distinct signal overlapping with the basal body (labeled using Centrin4–CFP) (Fig. 1Ef,g). In contrast, Rmnd5a was absent from the basal body of multi-ciliated cells of the skin, whereas it localized to centrioles and mid-bodies in non-ciliated epidermal cells (Fig. S1). Similar to the localization of GFP–RMND5A, we found clear endogenous RMND5A and TWA1 signals proximal to the axoneme with partial overlap in 60.7% (*n*=28) and 75.9% (*n*=29) of NIH-3T3 cells, respectively (Fig. 2A, RMND5A and TWA1), whereas ARMC8 and MKLN1 colocalized with the axoneme in 96.3% (*n*=27) and 93.1% (*n*=29) of all cells, respectively (Fig. 2A, ARMC8 and MKLN1). In cycling cells, we found TWA1 and RMND5A both at the mother and the daughter centriole in 87.1% (*n*=31) and 82.8% (*n*=29) of all cells, respectively (Fig. 2B). We conclude that RMND5A and other integral components of the GID complex are present at the basal body, at the axoneme in primary cilia and motile mono-cilia, and at the centrioles in cycling cells, suggesting that the GID complex has a function at the primary cilium. Specificities of the antibodies used either were tested by western blot analysis (for anti-RMND5A; Fig. S4) or had been established in a previous publication (anti-TWA1, anti-ARMC8 and anti-MKLN1; Maitland et al., 2019).

### Reduction of GID complex function interferes with SHH signaling

**Fig. 3.**
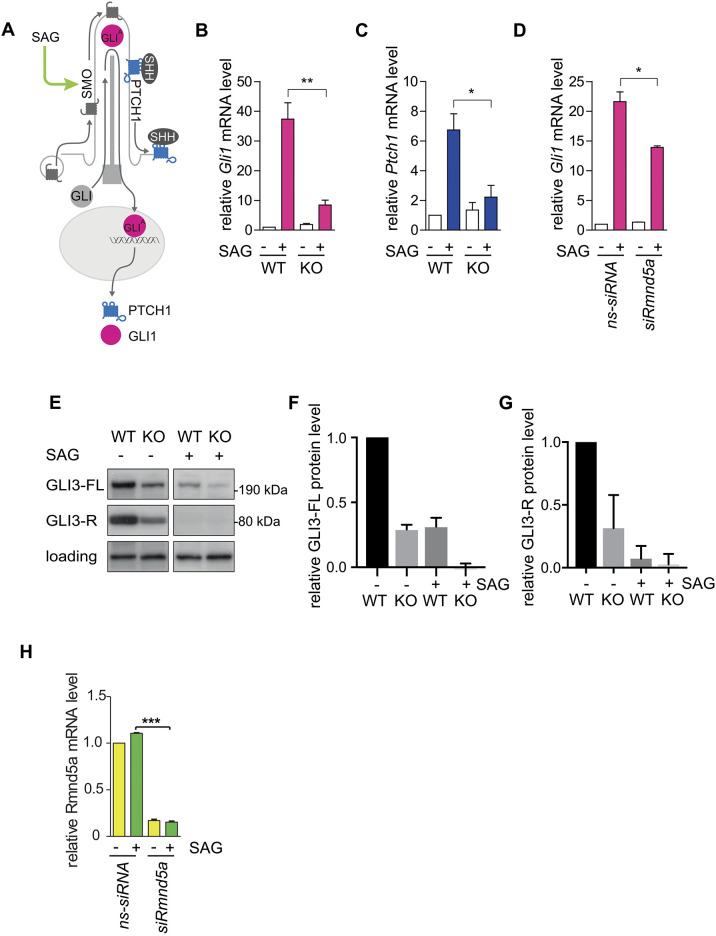
**GID deficiency interferes with SHH signaling.** (A) Schematic representation of primary cilium-dependent SHH signaling in the ‘on state’. After binding of SHH ligand to PTCH1, it relieves the inhibition of smoothened (SMO). Then, GLI transcription factors are activated (GLI^A^), turning on expression of downstream genes, such as *Gli1* and *Ptch1*. (B–D) qPCR of two SHH signaling markers (*Gli1* in B,D; *Ptch1* in C) during RMND5A KO (B,C) or knockdown (D). Cells were cultured under cilia-induced conditions (high glucose DMEM, 0.5% FCS) with or without SAG (100 nM) treatment for 24 h and harvested for further analysis. ns-siRNA, control siRNA; siRmnd5a, RMND5A knockdown. Mean±s.e.m., *n*=3. *P<0.05; **P<0.01 (two-tailed, unpaired Student's *t*-test). (E) Western blot of GLI3 (same blot and exposure time). WT and KO cells were cultured under cilia-induced conditions (high glucose DMEM, 0.5% FCS) with or without SAG (100 nM) treatment for 24 h and harvested. A protein that cross-reacted with the GLI3 antibody was used as a loading control. (F,G) Quantification of western blots as in E showing relative protein level of GLI3 full-length form (F) and of GLI3 repressor form (G) in NIH-3T3 WT and RMND5A KO cells. Signal intensities of all lanes were measured on the same western blot with same exposure times and are normalized to the WT −SAG condition. WT −SAG, GLI3-FL/GLI3-R=1.51; KO −SAG, GLI3-FL/GLI3-R=1.4. Data are mean±s.e.m. of *n*=3. (H) qPCR of *Rmnd5a* mRNA during RMND5A knockdown. Cells were cultured under cilia-induced conditions (high glucose DMEM, 0.5% FCS) with or without SAG (100 nM) treatment for 24 h and harvested. Data are mean±s.e.m. of *n*=3. ***P<0.001 (two-tailed, unpaired Student's *t*-test). Knockdown efficiency showed an 85% reduction of *Rmnd5a* mRNA.

Several essential components of the SHH signal transduction pathway, including the SHH receptor protein patched homolog 1 (PTCH1), are located in the primary cilium membrane or within the primary cilium (Fig. 3A). Defects in primary cilia correlate with malfunctioning SHH signal activation (Rohatgi et al., 2007), and consequently the pathway can be used to study a potential function of the GID complex at the basal body of primary cilia. The SHH signaling pathway can be activated by the addition of smoothened agonist (SAG), which induces the transcriptional upregulation of *Gli1* and *Ptch1* (Fig. 3A). Our data reveal that the relative transcript levels of *Gli1* and *Ptch1* are indeed several-fold upregulated upon SAG addition, indicating a functional SHH response in wild-type (WT) NIH-3T3 cells. In contrast, NIH-3T3 cells deficient in RMND5A (RMND5A knockout cells, KO) showed reduced *Gli1* and *Ptch1* transcriptional activation (Fig. 3B,C), indicating a strongly suppressed SHH signal transduction response. Similarly, WT cells treated with RMND5A-specific siRNA (Fig. 3D,H) showed a significant reduction of *Gli1* mRNA levels upon SAG treatment when compared to levels in the control (*P<0.05; Fig. 3D).

SHH-induced changes in gene transcription result in a large part from suppression of proteolytic processing of GLI3. In the absence of SHH signaling, the majority of full-length GLI3 (GLI3-FL) is processed by the proteasome to a truncated repressor form (GLI3-R) (Wang et al., 2000). The processing of GLI3-FL to GLI3-R requires accumulation of GLI3-FL at the ciliary tip, phosphorylation at different sites and functional intraflagellar transport (Laclef et al., 2015). Our results show that both WT and KO cells contain processed GLI3-R (Fig. 3E). The addition of SAG resulted in the rapid inhibition of GLI3 processing independent of a functional GID complex (Fig. 3E). Strikingly, the level of GLI3 protein in RMND5A-deficient cells was sharply reduced compared to that in WT cells (Fig. 3F,G). However, the ratio between GLI3-FL and GLI3-R under non-inducing conditions appeared unaffected (WT −SAG, GLI3-FL/GLI3-R=1.51; KO −SAG, GLI3-FL/GLI3-R=1.4). In conclusion, we find that GID complex function is necessary for a SHH and GLI1 response, although a low level of GLI3 processing is still possible.

### The reduction in SHH signaling is independent of the AMPK–MTOR signaling axis

**Fig. 4.**
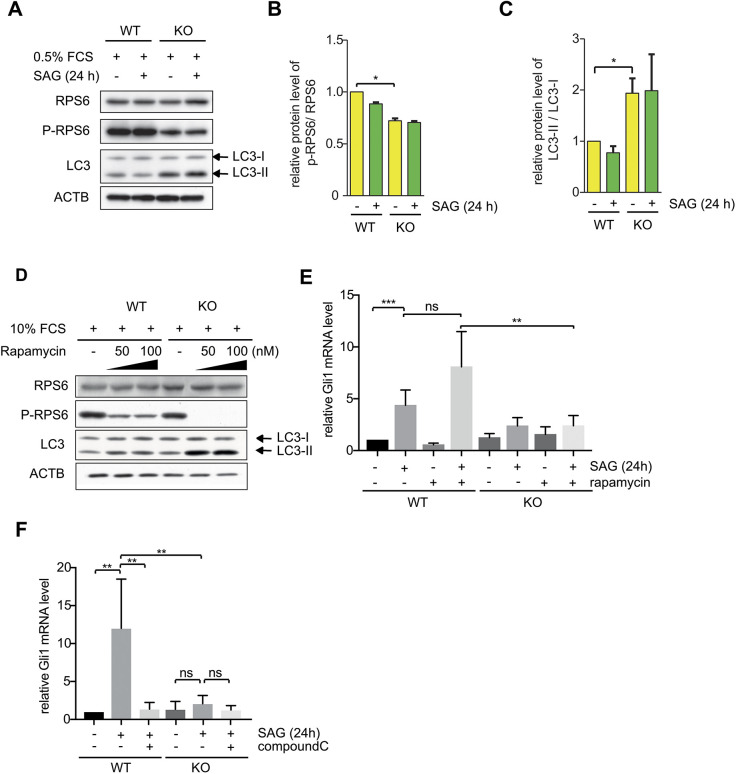
**Reduction of SHH signaling is independent of the AMPK–MTOR signaling axis.** (A) Western blot analysis of NIH-3T3 WT and RMND5A KO cell extracts with the markers of the MTOR–autophagy pathway RPS6, p-RPS6 and LC3. Cells were cultured under cilia-induced condition (high glucose DMEM, 0.5% FCS) with or without SAG (100 nM) treatment for 24 h. ACTB (β-actin) was used as a loading control. LC3, microtubule-associated protein 1A/1B-light chain 3; LC3-I, unlipidated cytosolic form of LC3; LC3-II, PEI-conjugated LC3 protein. (B,C) Quantification of western blotting as in A, showing the relative ratio between p-RPS6 and RPS6 (B), and between LC3-II and LC3-I (C). Data are mean±s.e.m., *n*=3. *P<0.05 (two-tailed unpaired Student's *t*-test). (D) Western blot analysis of NIH-3T3 WT and KO cell extracts with the markers of the MTOR–autophagy pathway RPS6, p-RPS6 and LC3. Cells were cultured under non-starvation conditions (high glucose DMEM, 10% FCS) with or without rapamycin (50 nM or 100 nM) treatment for 24 h. ACTB was used as a loading control. Blots are representative of three individual experiments. (E) qPCR of relative mRNA levels of the SHH signaling marker *Gli1* during rapamycin treatment. WT and KO cells were cultured under cilia-induced condition (high glucose DMEM, 0.5% FCS) with SAG (500 nM) and rapamycin (1 μM) treatment for 24 h as indicated. Mean±s.e.m., *n*=5. **P<0.01; ***P<0.001; ns, not significant (two-tailed unpaired Student's *t*-test). (F) qPCR of relative mRNA levels of the SHH signaling marker *Gli1* during compound C treatment. WT and KO cells were cultured under cilia-induced condition (high glucose DMEM, 0.5% FCS) with SAG (100 nM) and compound C (10 μM) treatment for 24 h as indicated. Mean±s.e.m., *n*=6. **P<0.01; ns, not significant (two-tailed unpaired Student's *t*-test).

The GID complex regulates AMPK activity (Liu et al., 2019) and the SHH response (Fig. 3). On the one hand, a series of studies has shown that the SHH pathway affects autophagy via AMPK (Xiao et al., 2015; Xu et al., 2014). On the other hand, it has been reported that AMPK signaling negatively regulates SHH signaling (Di Magno et al., 2016; Li et al., 2015). Consequently, we were curious to investigate whether increased AMPK activity in KO cells is influenced by SHH signaling. In this context, we examined MTOR activity and autophagic flux upon activation of SHH signaling in WT and KO cells by measuring the phosphorylation status of the ribosomal protein S6 (RPS6) or the level of phosphatidylethanolamine (PEI)-modified MAP1LC3A (LC3-II) (Fig. 4A) (Klionsky et al., 2021). As previously reported, the relative level of phosphorylated RPS6 (p-RPS6) was significantly reduced (Fig. 4A,B) and LC3-II levels were significantly increased in RMND5A KO cells; however, this was independent of SHH signaling (Fig. 4A,C) (Liu et al., 2019). Taken together, these results show that the induction of SHH signaling does not affect AMPK–MTOR activity in WT and KO cells.

The treatment of WT cells with the MTOR inhibitor rapamycin resulted in a severe reduction of p-RPS6 and a slight increase in LC3-II levels. The effects of rapamycin were strongly enhanced in RMND5A-deficient cells (Fig. 4D). Since rapamycin acts on MTOR downstream of AMPK, we further examined whether the stimulation of SHH signaling, monitored by *Gli1* gene activation, was altered upon blocking MTOR or AMPK. In WT cells, rapamycin treatment did not significantly alter SHH activation by SAG (Fig. 4E). Dorsomorphin (compound C) is a selective inhibitor of AMPK (Zhou et al., 2001). The treatment of WT cells with compound C resulted in a significant reduction of *Gli1* gene activation upon induction of SHH signaling (Fig. 4F). However, in KO cells, the lack of SHH activation upon SAG treatment was not compensated by inhibition of either AMPK or MTOR (Fig. 4E,F). In summary, our data suggest that the GID complex regulates AMPK activity independently of its function in SHH signaling.

### Reduction of GID complex function results in aberrant protein homeostasis of SHH signaling components in primary cilia

**Fig. 5.**
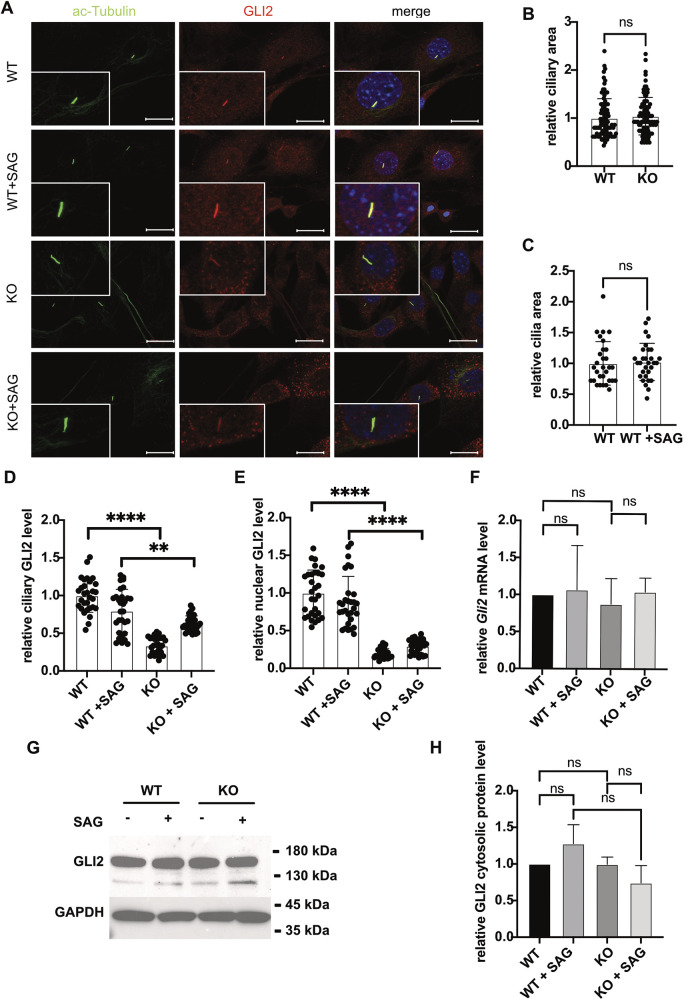
**Reduction of GID complex function results in aberrant protein homeostasis in primary cilia.** (A) Representative microscope images of GLI2 localization. WT and KO cells were treated with cilia-inducing medium (high-glucose DMEM with 0.5% FCS) for 24 h with or without SAG and then fixed. Axonemes were stained with anti-acetylated TUBA4A (ac-tubulin) antibody (green); anti-GLI2 antibody (red) was used to visualize GLI2 protein, and DAPI (blue) was used to stain DNA. Inset images show magnification of a primary cilium. Scale bars: 20 μm. (B) Relative ciliary area of WT and KO. Relative cilia area was measured in the area demarcating the axoneme (green) in WT and KO cells, and the WT average set to 1. Mean±s.e.m.; *n*=90 (WT), *n*=90 (KO). ns, *P*>0.05 (two-tailed, unpaired Student's *t*-test). (C) Relative ciliary area of WT and WT+SAG. Relative cilia area was measured in the area demarcating the axoneme (green) in WT and WT+SAG cells, with the WT average set to 1. Mean±s.e.m.; *n*=30 (WT), *n*=30 (KO). ns, *P*>0.05 (two-tailed, unpaired Student's *t*-test). (D) Relative ciliary GLI2 protein level. GLI2 (red) and ac-Tubulin (green) fluorescence intensity was measured in the area demarcating the axoneme (green) in WT and KO cells with or without SAG (100 nM). Intensity was normalized to the level of ac-tubulin. Mean±s.e.m.; *n*=30 (WT), *n*=30 (WT+SAG), *n*=30 (KO), *n*=30 (KO+SAG). ****P<0.0001; **P=0.0038 (two-tailed, unpaired Student's *t*-test). (E) Relative nuclear GLI2 protein level. GLI2 fluorescence intensity (red) was measured in the area demarcating the nucleus (blue) in WT and KO cells with or without SAG (100 nM) and normalized to the signal intensity of DAPI. Mean±s.e.m.; *n*=30 (WT), *n*=30 (WT+SAG), *n*=30 (KO), *n*=30 (KO+SAG). ****P<0.0001 (two-tailed, unpaired Student's *t*-test). (F) qPCR of relative *Gli2* levels in WT and KO cells. Cells were cultured under cilia-induced condition (high glucose DMEM, 0.5% FCS) with or without SAG (100 nM) treatment for 24 h, as indicated, and harvested for further analysis. Mean±s.e.m., *n*=6. ns, *P*>0.05 (two-tailed, unpaired Student's *t*-test). (G) Western blot analysis of cytosolic GLI2 in WT and KO cells. Cells were cultured under cilia-induced condition (high glucose DMEM, 0.5% FCS) with or without SAG (100 nM) treatment for 24 h. GAPDH was used as a loading control. (H) Quantification of western blot signals as in G, showing the relative ratio between cytosolic GLI2 and GAPDH. Mean±s.e.m., *n*=3. ns, *P*>0.05 (two-tailed, unpaired Student's *t*-test).

Spatially timed protein localization of SHH signaling components is critical for SHH signaling and cilia function. To test whether protein localization is affected in GID-deficient cells, we investigated the localization of important SHH signaling components in WT and KO NIH-3T3 cells (Fig. 5A; Fig. S2). Proteins, including the SHH receptor protein PTCH1, the transcription factors GLI1 and GLI2, and SUFU, were properly located at or in the primary cilium. First, we quantified the relative area of cilia, comparing WT cells with KO cells (Fig. 5B), as well as WT versus WT upon SAG induction (Fig. 5C). In both settings, the relative area of the cilium was not significantly altered. Second, we quantified the relative levels of SHH signaling components in the cilium (Fig. 5D; Fig. S2B,D,G) and in the nucleus (Fig. 5E; Fig. S2E). Quantification of protein levels in WT and KO cells showed a strong reduction of GLI2 (Fig. 5D) and PTCH1 (Fig. S2A,B) in the axoneme of KO cells, whereas other proteins, including SUFU (Fig. S2F,G) and GLI1 (Fig. S2C,D), were not affected and did not show a reduction of protein in the cilium. A reduction in GLI2 and GLI1 was also evident in the nucleus (Fig. 5E; Fig. S2E) but appeared to be independent of SAG induction (Fig. 5E). Moreover, the reduction in GLI2 protein in the cilium and the nucleus appeared to be independent of transcription (Fig. 5F) and cytosolic GLI2 protein (Fig. 5G). The amount of cytosolic GLI2 protein and *Gli2* transcript was not affected by loss of GID function (Fig. 5F,H). This suggests a selective regulation of ciliary SHH signaling protein homeostasis by the GID complex, which is likely dependent on protein transport to the cilium and/or import into the cilium. Thus, the sharp reduction in levels of some SHH components in the cilium is probably the underlying reason for SHH signal transduction defects in KO cells.

### Genes encoding GID complex subunits are expressed in ciliated organs, and loss of GID function causes ciliopathy-like phenotypes in *X. laevis*

**Fig. 6.**
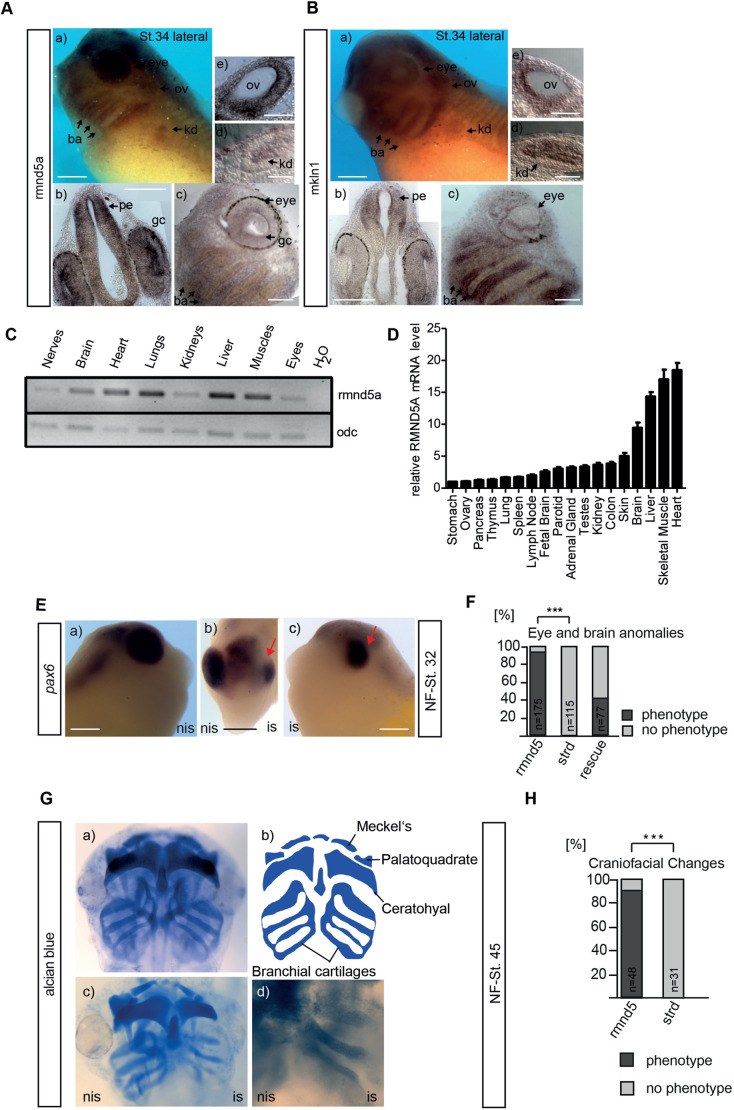
**GID genes are expressed in ciliated organs during *X. laevis* development.** Spatial analysis of expression of GID complex subunits *rmnd5a* (A) and *mkln1* (B) in *X. laevis.* WMISH of WT *X. laevis* embryos at developmental stage 34 (St. 34; lateral views in Aa and Ba) and the corresponding transverse (b,e,d) and sagittal (c) sections (ov, otic vesicle; pe, prosencephalon; ba, branchial arches; kd, pronephric kidney; gc, ganglion cells). Images are representative of three experiments. Images in Ab and Bb are the result of tiling multiple fields of view. Scale bars: 300 μm in Aa–c and Ba–c; 100 μm in Ad, Ae, Bd and Be. (C) RT-PCR analysis of *rmnd5a* expression in distinct tissues of *X. laevis*. RT-PCR of *odc1* (*odc*) is shown as an RNA input control (bottom). Data are representative of three experiments. (D) Quantification of relative mRNA levels of human *RMND5A* in various tissues. cDNA was reverse transcribed from the indicated RNA samples. β-actin (*ACTB*) levels were used as the qPCR internal control. *RMND5A* mRNA of different tissues is compared to its corresponding housekeeping gene (*ACTB*) in percent. In most tissues, *RMND5A* is expressed in an amount equivalent to more than 5% of the housekeeping gene. Mean±s.e.m., *n*=3. (E–H) Rmnd5a alteration is associated with ciliopathy-like phenotypes. (E) *rmnd5a*-mo-injected embryos (NF-St. 32, NF stage 32; is, injected side; nis, non-injected side) were used for *in situ* hybridization with the *pax6* marker. The red arrow in images b and c indicates the eye of the injected side of the morpholinos. Scale bars: 300 μm. (F) Quantitative representation of *rmnd5a*-mo (rmnd5), negative control standard-mo (strd) and rescue (co-injection with *rmnd5a*-mo and synthetic Rmnd5a-encoding RNA) phenotypes, presented as a bar graph (embryos with phenotype as a percentage of the total number of embryos scored; black, phenotype; gray, no phenotype). *n*=number of injected embryos analyzed for the respective marker. ***P≤0.001 (chi-square test). (G) Analysis of the formation of head cartilage by Alcian Blue cartilage staining in swimming tadpoles at NF stage 45 (NF-St. 45). Meckel's cartilage, ceratohyal cartilage, branchial cartilage and basihyal cartilage is shown in control standard-mo-injected (a) and *rmnd5a*-mo-injected (c,d) tadpoles (is, injected side; nis, non-injected side). A schematic of WT cartilage formation is shown in image b. (H) Quantitative representation of craniofacial changes in *rmnd5a*-mo-injected and standard-mo-injected tadpoles, with phenotypes displayed as a bar graph (embryos with a phenotype as a percentage of the total number scored; black, phenotype; gray, no phenotype). *n*=number of injected embryos analyzed. ***P≤0.001 (chi-square test).

We have previously shown that expression of the GID complex subunit *rmnd5a* is restricted mainly to head structures particular to the prospective prosencephalon (Pfirrmann et al., 2015). Here, we more closely examined *rmnd5a* expression, with a focus on ciliated tissues. In the developing *X. laevis* tadpole, ciliated tissues include the olfactory placode/pit, the lining of the brain ventricles, the otic vesicle, the pronephric tubule, branchial arches and the skin (Walentek and Quigley, 2017). Whole-mount *in situ* hybridization (WMISH) of tadpoles at Nieuwkoop and Faber (NF) stage 34 revealed that *rmnd5a* transcripts are present in the ganglion cell layer of the eye (Fig. 6Aa,c; gc) and the prospective prosencephalon (Fig. 6Ab,c; pe). Rmnd5a transcripts were also observed in other ciliated tissues, prominently the otic vesicle (Fig. 6Aa,e; ov), the branchial arches (Fig. 6Aa,c; ba), and throughout the pronephric tubule (Fig. 6Aa,d; kd). Interestingly, *rmnd5a* expression increased towards the dorsal territories of the prospective prosencephalon and the lining of the ventricle, a region of the brain with motile cilia required for cerebrospinal fluid flow in the ventricular system (Hagenlocher et al., 2013) (Fig. 6Ab; pe). To exclude that *rmnd5a* expression in these tissues appears independently of other GID complex members, we investigated the expression of Mkln1 (also known as Gid7 in *X. laevis*) as a further GID complex subunit. Strikingly, *mkln1* was expressed in a very similar pattern to that of *rmnd5a* (Fig. 6B). Of note, *mkln1* expression was also increased in the dorsal part of the prospective prosencephalon and mesencephalon, and the signal appeared to be more intense towards the lining of the dorsal ventricle. Thus, several GID subunits are expressed in ciliated organs of the developing organism in a similar spatial pattern. This prompted us to test whether *rmnd5a* expression is restricted to the same organs in the adult using semi-quantitative reverse transcription PCR (RT-PCR) to assay total RNA from adult *X. laevis* organs. Transcripts of *rmnd5a* were detected in all investigated organs. We found the most robust *rmnd5a* expression in liver, muscle, lung, heart and brain, and the lowest expression in kidney, nerve and eye (Fig. 6C). Similar to expression in *X. laevis*, the *RMND5A* transcription pattern in 18 human organs and tissues showed the highest *RMND5A* expression in the heart, muscle, liver and brain (Fig. 6D). Taken together, these data suggest that *RMND5A* expression in ciliated organs could be evolutionarily conserved.

To test whether *rmnd5a* morphants develop ciliopathy and/or SHH loss-of-function-related phenotypes, we suppressed the function of Rmnd5a using antisense oligonucleotides (morpholino, *rmnd5a*-mo). The microinjection of *rmnd5a*-mo into one blastomere at the two-cell stage resulted in developmental alterations, including brain anomalies reminiscent of occipital encephalocele and severe eye anomalies reminiscent of microphthalmia (Fig. 6E,F; Table S5) (Vogel et al., 2012). Primary cilia and SHH signaling are also involved in the development of cartilaginous anlagen of the head (Abdelhamed et al., 2013; Lee and Gleeson, 2011; Tao et al., 2020). Craniofacial development is initiated by cranial neural crest cells that migrate from the dorsal neural tube into a series of branchial arches to give rise to the Meckel's and palatoquadrate cartilages, the ceratohyal cartilages and the branchial cartilages (Baltzinger et al., 2005) (Fig. 6G,b). Craniofacial defects can be visualized by Alcian Blue staining in the developing tadpole (Abramyan, 2019). We performed Alcian Blue staining of *rmnd5a* morphants at NF stage 45 to follow craniofacial development. Depletion of *rmnd5a* led to shortened Meckel's, ceratohyal, branchial and palatoquadrate cartilage (Fig. 6Gc, is: Fig. 6H). An organ also often affected in ciliopathies is the kidney, and patients with ciliopathies can develop polycystic kidneys. We investigated whether the development of the pronephros was impaired in *rmnd5a* morphants (Fig. S3). Following microinjection of *rmnd5a-*mo, the pronephric kidney appeared mostly unaffected, including all areas of the proximal tubule, such as the intermediate tubule, the distal tubule and the collecting duct (Fig. S3A). This was also evident in the corresponding sections of the pronephros (Fig. S3Ba′,b′). In summary, the *rmnd5a* morphant phenotype resembles several alterations known in ciliopathies, with particular overlap in the more severe spectrum of ciliopathies (Table S5).

### Loss of GID function affects patterning events of the prospective forebrain

**Fig. 7.**
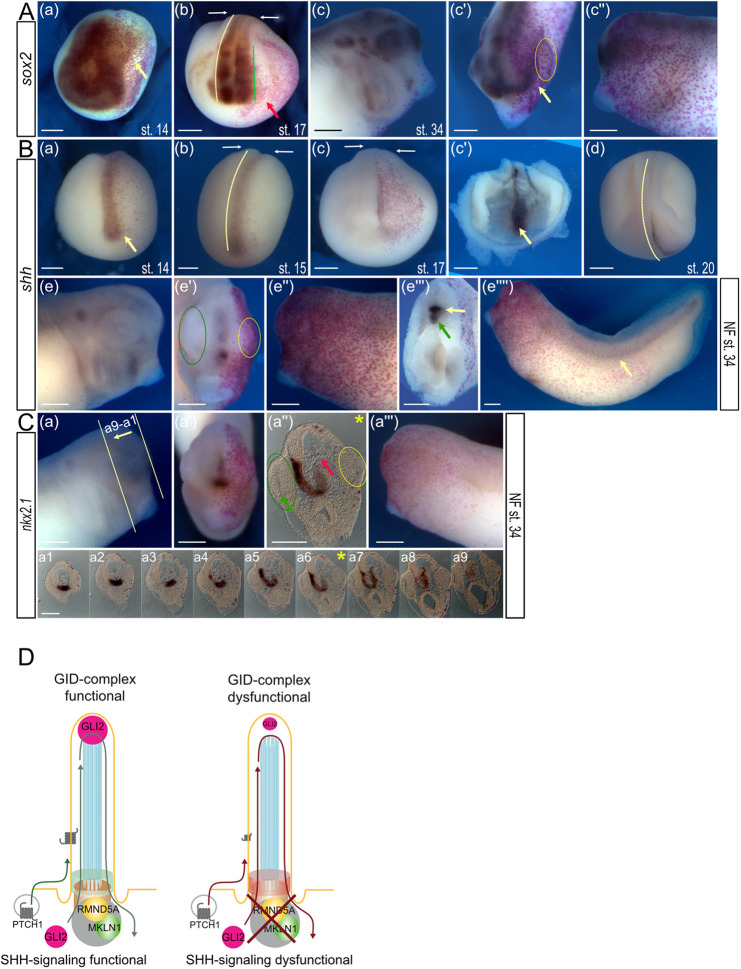
**Suppression of *rmnd5a* function interferes with neural patterning.** (A–C) Embryos injected with *rmnd5a*-mo were used for *in situ* hybridization with (A) *sox2* (pan-neural marker), (B) *shh* (axial mesoderm and SHH target gene in the floor plate) and (C) *nkx2.1* (marker of the ventral forebrain) at the indicated NF stages (st.; st. 14 in Aa, Ba; st. 15 in Bb; st. 17 in Ab, Bc, Bc′; st. 20 in Bd; st. 34 in Ac–Ac″, Be–Be″″ and C). Injected sides are shown on the right of the images (red color) and in Ac″, Ae″, Ae″″ and Ca‴, non-injected sides are shown on the left of the images and in Ac, Ae and Ca. Images show anterior views (Aa, Ab, Ac′, Ba-Bc, Be′, Be‴, Cb), lateral views (Ac, Ac″, Be, Be″, Be″″, Ca, Ca‴) or superior views (Bd). Expression of *sox2* at NF stage 14 is shown in image Aa (anterior view; *n*=29/31); the yellow arrow indicates less patterned *sox2* expression. The white arrows in images Ab, Bb and Bc indicate delayed development of the neural tube and smaller hemisphere. Yellow dashed lines in images Ab, Bb and Bd highlight sickle-shaped curvature towards the injected side. Green dashed line in image Ab indicates less patterned *sox2* expression of the injected side within the presumptive brain region. Red arrow in image Ab indicates alleviated formation of the eye vesicle. Yellow arrow in image Ac′ marks the shifted olfactory anlage, and the oval indicates the remnant of the larval eye (visible in Ac–c″). (B) Suppression of *rmnd5a* function results in almost normal *shh* expression domains. Yellow arrows indicate the prechordal plate; image Bc′ shows an inner view of an opened larva at NF stage 17. Yellow dashed line in image Bd indicates bending of the larva. Reduction of forebrain and eye development can be seen upon Rmnd5a-knockdown (yellow dashed oval) compared to the non-injected side (green dashed oval) in image Be′. Green arrow in Be‴ shows primary expression of *shh* in the notochord; yellow arrow in Be‴ and Be″″ shows secondary domain of *shh* expression in the floor plate of the neural tube. (C) Expression of *nkx2.1* in NF stage 34 embryos (images a,a′,a‴) and 20 µm vibratome sections (Ca1–a9, positions as indicated in image Ca; frontal plane shown in image Ca″). In image Ca″, the green arrow indicates the prospective lens on the non-injected side, the red arrow indicates a reduction of *nkx2.1* expression on the injected side, the green oval indicates the prospective eye on the non-injected side, and the yellow oval indicates the prospective eye on the injected side. Asterisk indicates frontal section of the anterior head shown in Ca″ and Ca6. Ca and Ca‴ show lateral views of the non-injected side and injected side of the embryo, respectively. Scale bars: 300 μm. (D) Model of the primary cilium and a proposed function of the cilia-localized GID complex in the regulation of SHH protein homeostasis in the cilium. Left: primary cilium with a functional cilia-localized GID complex and functional SHH signaling response. Protein homeostasis of SHH components in the cilium is maintained (green). Right: primary cilium with aberrant protein homeostasis, reduction of SHH signaling components – namely GLI2 and PTCH1 – in the cilium and dysfunctional SHH signaling response without a functional GID complex (red).

Microinjection of *rmnd5a*-mo into one blastomere of *X. laevis* eggs at the two-cell stage resulted in developmental alterations, including brain anomalies and severe eye anomalies (Fig. 6E,F; Table S5). To investigate the underlying molecular reasons for these phenotypes, we suppressed the function of Rmnd5a using antisense oligonucleotides (*rmnd5a*-mo) and followed expression of *sox2* (pan-neural marker), *shh* (axial mesoderm and SHH target gene in the floor plate) and *nkx2.1* (marker of the ventral forebrain) by WMISH (Fig. 7). Suppression of *rmnd5a* function did not interfere with primary neural induction in the neural plate territory as monitored by *sox2* expression at NF stage 14 (anterior view; *n*=29/31; Fig. 7Aa). Though the neural tube appeared to close almost normally, the development of the injected side of the *rmnd5a* morphants appeared slightly delayed (white arrows in Fig. 7Ab,Bb and Bc) and resulted in a smaller hemisphere with a sickle-shaped curvature towards the injected side (yellow dashed line in Fig. 7Ab,Bb and Bd, anterior view). In addition, *sox2* expression on the injected side was less patterned within the presumptive brain region (green dashed line in Fig. 7Ab). Moreover, the formation of the eye vesicle was alleviated (red arrow in Fig. 7Ab). In tadpoles (NF stage 34), the development of the forebrain and eyes appeared hindered, because the head of the injected side was smaller as evident by the shift of the olfactory anlage (yellow arrow in Fig. 7Ac′). The yellow arrow in Fig. 7Ac′ marks the shifted olfactory anlage, and the oval in the same image marks the remnant of the larval eye (Fig. 7Ac–c″). In contrast, the expression domains of *shh* appeared almost normal (Fig. 7Ba–d). During neurulation at NF stages 14, 15 and 17, *shh* transcription within the axial mesoderm that later forms the notochord and the prechordal plate was not altered in time and space (Fig. 7Bc′ shows an inner view of an opened larva at NF stage 17, the yellow arrow indicates the prechordal plate). However, bending of the larvae was evident (yellow dashed line in Fig. 7Bd). At tadpole stage (NF stage 34), the morphants showed a clear reduction of forebrain and eye development [compare yellow (*rmnd5a*-mo) and green (non-injected side) dashed ovals in Fig. 7Be′]. Similarly, not only did the primary expression of *shh* in the notochord appear unchanged upon *rmnd5a* knockdown (green arrow, Fig. 7Be‴), even the secondary domain of *shh* expression in the floor plate of the neural tube was unaffected (yellow arrow, Fig. 7Be‴). In fact, unlike the head region, the trunk and tail of the *rmnd5a* morphants developed normally (Fig. 7Be″″). Transcripts of *nkx2.1*, a marker of the ventral forebrain, were monitored to illustrate patterning events of the prospective forebrain, which highly depend on *shh* signaling. The expression of *nkx2.1* was clearly reduced on the injected side (Fig. 7Ca″ and Fig. 7Ca1–a9). To illustrate the affected *nkx2.1* expression upon *rmnd5a* knockdown, all frontal sections of the anterior head are presented (Fig. 7Ca1–a9). Taken together, our observations indicate that the suppression of *rmnd5a* function does not affect shh-independent primary neural induction, but strongly affects the proper formation of the forebrain, likely due to changes in SHH signaling-dependent patterning events.

## DISCUSSION

The primary cilium contains diverse receptors required to perceive multiple stimuli, including morphogens, fluid flow and light (Zimmerman and Yoder, 2015). Several recent observations link primary cilia function with cellular and organismal energy homeostasis (Boehlke et al., 2010; Orhon et al., 2016; Pampliega et al., 2013). This suggests the existence of a molecular mechanism that perceives energy-dependent intracellular and/or extracellular signals at the cilium. The idea of such cilia-dependent processes regulating energy homeostasis is supported by the phenotypic spectrum of a subgroup of ciliopathies. BBS and Almström syndrome are accompanied by symptoms of dysregulated energy homeostasis, such as morbid obesity and type 2 diabetes (Guo et al., 2016; Lee et al., 2015). Several phenotypic aspects of BBS overlap with those found in Meckel–Gruber syndrome (Karmous-Benailly et al., 2005), a lethal autosomal recessive congenital disorder that leads to enlarged kidneys with numerous fluid-filled cysts, occipital encephalocele and polydactyly (Hartill et al., 2017). The spectrum of phenotypic anomalies that we observed in our model organism *X. laevis* includes structural brain anomalies and severe defects in craniofacial development (Table S5). These phenotypes overlap with the classic triad of Meckel–Gruber syndrome that includes cystic renal disease, polydactyly and a central nervous system malformation, most commonly occipital encephalocele (Wright et al., 1994). Interestingly, a patient with a giant occipital encephalocele and craniofacial anomalies carries a partial duplication of the *RMND5A* gene (Vogel et al., 2012). The alterations observed in *rmnd5a* morphants and in this patient resemble those in Meckel–Gruber syndrome. Thus, we speculate that the Meckel–Gruber-like phenotypes might be caused by a dysfunctional GID complex with defects in cilia-dependent signaling processes, including defects in SHH signaling.

The SHH signaling pathway is among the best studied signaling systems coordinated by the primary cilium (Song et al., 2018). In the absence of SHH ligand, GLI2 and GLI3 transcription factors are sequestered in the cytoplasm through interaction with SUFU (Pietrobono et al., 2019). GLI3 is processed to the truncated GLI3-R repressor form by the proteasome at the base of the cilium and then moves to the nucleus to keep target gene transcription in the off state (Pan et al., 2006). In response to SHH pathway activation, processing of GLI3 is blocked, resulting in the activation of the full-length GLI3-FL form, which translocates into the nucleus to induce target gene transcription (Kim et al., 2009). Elucidating the molecular mechanisms by which the GID complex interferes with SHH signaling provides the first understanding of this process. Our data show that cells with compromised GID complex activity display impaired SHH signaling with an almost complete lack of transcriptional *Gli1* upregulation by SAG. However, KO cells perform GLI3 processing – although on a much lower level than in WT cells and with an unaffected GLI3-FL to GLI3-R ratio – and most importantly, the pathway can be activated by SAG, resulting in the complete removal of GLI3-R (Fig. 3). This demonstrates that in GID complex-deficient cells, activation of the G-protein-coupled receptor smoothened by SAG can still force the processing of remaining GLI3 protein, but the resulting transcriptional signal is too weak and thus is not able to amplify the transcriptional response. Furthermore, this suggests that the transition zone-located protein retinitis pigmentosa GTPase regulator-interacting protein 1-like (RPGRIP1L) fully activates the specialized cilium-regulated proteasome, which has previously been shown to be required for GLI3 processing (Gerhardt et al., 2015). In 2005, Haycraft and colleagues showed that the GLI proteins (GLI1, GLI2 and GLI3) localize at the distal tips of primary cilia without any stimulation of SHH signaling (Haycraft et al., 2005). Subsequently, several studies have revealed an accumulation of the GLI protein at the ciliary tip upon stimulation of SHH signaling (Chen et al., 2009; Massa et al., 2019; Moon et al., 2014; Wen et al., 2010; Yang et al., 2015; Yoshida et al., 2020). However, several reports have described a presence of GLI2 and GLI3 in the entire cilium without SHH stimulation (Clement et al., 2009; Egeberg et al., 2012; Emechebe et al., 2016; Kiprilov et al., 2008), reflecting transport of the GLI proteins from the ciliary base to the distal tip and vice versa (Eguether et al., 2014; Kim et al., 2009; Qin et al., 2011). In this context, it has been demonstrated that the localization of GLI3 along the entire cilium is not changed by SAG treatment (Emechebe et al., 2016). In our study, SAG treatment did not result in an accumulation of GLI2 at the ciliary tip but, importantly, it led to a consistent and significant upregulation of target gene expression, demonstrating robust pathway activation upon SAG treatment. Despite the successful induction of SHH signaling by SAG, our experiments revealed that the ciliary amount of GLI2, the predominant activator of SHH signaling, was significantly reduced in RMND5A KO cells (Fig. 5A,B), reflecting impaired SHH signaling. In addition to these *in vitro* studies, we performed *in vivo* experiments in *X. laevis*. To knockdown Rmnd5a translation, an antisense morpholino was injected into one cell of two-cell-stage embryos, together with synthetic RNA encoding nuclear β-galactosidase to mark the injected side of the larvae visible as red nuclear staining. As SHH is essential for the development of the forebrain, we analyzed the expression of *shh* and its target gene *nkx2.1* in this process. In the notochord and the floor plate of the injected side, *shh* expression appeared unaltered (Fig. 7B). However, the expression of *nkx2.1* in the ventral forebrain was severely decreased, revealing impaired SHH signaling in *rmnd5a*-deficient *X. laevis* forebrains. Interestingly, SHH signals from the notochord are able to induce SHH expression in the floor plate in the absence of Rmnd5a, although SHH signaling is impeded. A similar situation has been reported in the neural tube of *Ft*/*Ft* homozygous mutant mouse embryos at embryonic day 10.5, in which the induction of *Shh* expression in the floor plate is successful, but SHH target expression in the ventral neural tube is hampered (Götz et al., 2005). In these embryos, six genes – *Irx3*, *Irx5*, *Irx6*, *Fto*, *Fts* (also known as *Aktip*) and *Rpgrip1l* – are deleted (Peters et al., 2002). One of the products of these genes, RPGRIP1L, localizes to the ciliary transition zone and is involved in SHH signal transduction (Gerhardt et al., 2015; Vierkotten et al., 2007; Wiegering et al., 2019). Importantly, morphogenesis of the forebrain and the eyes is strongly perturbed in *Ft*/*Ft* mouse embryos (Anselme et al., 2007) and resembles the phenotype we observed for the *rmnd5a*-deficient side in *X. laevis* morphants. We reason that the forebrain and eye phenotype of *Ft*/*Ft* embryos might be caused by the loss of RPGRIP1L, since *Rpgrip1l*-negative mouse embryos display defects in forebrain and eye development as well (Besse et al., 2011; Delous et al., 2007; Vierkotten et al., 2007). Remarkably, mutations in *RPGRIP1L* result in severe human ciliopathies (Delous et al., 2007). All these findings point to a close relationship between the GID complex, SHH signaling, cilia and ciliopathies. Concerning our *in vitro* and *in vivo* data on SHH signaling, we hypothesize that the lack of target gene activation in the absence of RMND5A is a result of the reduced amount of SHH signaling components in the cilium.

Recently, we have demonstrated that the GID complex negatively regulates AMPK (Liu et al., 2019). Additionally, it has been shown that the SHH pathway affects autophagy via AMPK (Xiao et al., 2015; Xu et al., 2014). However, AMPK signaling is also able to regulate SHH signaling negatively (Di Magno et al., 2016; Li et al., 2015). To test for a potential dependency of the effects on AMPK and SHH signaling in RMND5A-negative cells, we used different drugs and reached the conclusion that the GID complex regulates SHH and AMPK signaling independently of each other. In this context, we administered the AMPK inhibitor compound C to SAG-treated WT cells and detected a decreased SHH target gene expression (Fig. 4F), indicating that AMPK positively regulates SHH signaling in NIH-3T3 cells. Since the negative regulatory effect of AMPK on SHH signaling has been demonstrated previously in NIH-3T3 cells (Di Magno et al., 2016; Li et al., 2015), it is unlikely that AMPK has a cell-type-specific effect on SHH signaling. Potentially, the observed reduction of SHH target gene expression, caused by treatment with compound C, could be a consequence of a side effect of compound C. Kwon et al. have described an inhibitory and AMPK-independent effect of compound C on platelet-derived growth factor receptor β (PDGFRβ) (Kwon et al., 2013). It is already known that PDGFRβ signaling activates SHH signaling, resulting in increased expression of the SHH target gene *Gli1* (Fingas et al., 2011). Accordingly, the reduced SHH target gene expression in WT NIH-3T3 cells treated with a combination of SAG and compound C could be triggered by the inhibition of PDGFRβ.

Regarding the relationship between the GID complex and primary cilia, there are various exciting links. Interestingly, several proteins encoded by genes that have been identified to be associated with Meckel–Gruber syndrome form large complexes that partly localize to the basal body (Sang et al., 2011). Consistent with an essential function at the basal body, we detected RMND5A and other components of the GID complex at the basal bodies and in the ciliary axoneme in NIH-3T3 cells, as well as in the basal bodies of motile mono-cilia in the epithelium of the GRP of *X. laevis* (Figs 1,2; Fig. S1) (Vick et al., 2009). At this stage, it is unknown whether the differing distribution of GID complex subunits can be explained by a function independent of the GID complex or by different subunit compositions that localize either to the basal body or the axoneme.

Various protein quality control systems in the cell remove misfolded proteins and prevent their accumulation in different cellular compartments, including the cytosol, nucleus, endoplasmic reticulum and others. Major strategies of these quality control systems involve protein refolding, degradation or sequestration. In many cases, polyubiquitylation of a target protein results in its specific degradation by the 26S proteasome (Pickart and Eddins, 2004). Recent evidence suggests that protein homeostasis at the primary cilium is maintained by the cilia-regulated proteasome (Gerhardt et al., 2015, 2016; Struchtrup et al., 2018). Furthermore, several recent publications describe a function of the ubiquitin–proteasome system (UPS) in the context of cilia function or ciliogenesis, suggesting a crucial role of ubiquitin ligases and protein degradation at the cilium, and especially at the transition zone and basal body (Lv et al., 2021; Wiegering et al., 2019). As a prominent example, TRIM32 is a member of the tripartite motif ubiquitin ligase family, and mutations in the *TRIM32* gene cause BBS (Chiang et al., 2006).

In conclusion, the cilia-localized GID complex is a crucial component of the UPS and is important for a functional SHH signaling response. We hypothesize that the cilia-localized GID complex functions in the regulation of selected protein homeostasis at and/or in primary cilia. In the future, it will be interesting to investigate whether members of the GID complex cause human ciliopathies associated with defects in SHH signal transduction.

## MATERIALS AND METHODS

### Organisms, cells and maintenance

*X. laevis* frogs were obtained from a commercial supplier (NASCO, USA). Production and rearing of embryos were performed as described previously (Hollemann et al., 1998), and embryos were maintained at 15°C and staged according to Nieuwkoop and Faber (1967). All procedures were performed according to guidelines set by the German animal use and care laws (Tierschutzgesetz) and approved by the German state administration Saxony-Anhalt (Projekt/AZ: 42502-3-600 MLU). NIH-3T3 cells (ATCC, CRL-6442; RRID: CVCL_0594) were maintained in Dulbecco's modified Eagle's medium (DMEM) supplemented with 10% (v/v) fetal calf serum (FCS) and 4500 mg/l glucose (high concentration) if not mentioned otherwise. An RMND5A knockout cell line was constructed as described previously (Liu et al., 2019), and all cells were tested for contamination by PCR on a regular basis. SAG (Sigma-Aldrich, SML1314) was applied at a concentration of 100 nM, rapamycin (Sigma-Aldrich, R0395) was applied at a concentration of 1 μM, and compound C (Sigma-Aldrich, 171261) was applied at a concentration of 10 μM, if not described otherwise.

### Capped mRNA and morpholino injections

Capped *rmnd5a* mRNA was generated using the mMESSAGE mMACHINE kit (Ambion, Austin, TX). Not1-linearized pTP251 (Pfirrmann et al., 2015) was used as a template for SP6 transcription, and 5 nl of capped mRNA (∼2.5 ng) was injected into one blastomere of a two-cell-stage embryo together with *rmnd5a* morpholino as previously described (Pfirrmann et al., 2015). The standard negative control morpholino (standard-mo) was purchased from Gene Tools.

### Whole-mount *in situ* hybridization

To analyze the spatial expression of *rmnd5a* and *mkln1* during *X. laevis* embryogenesis, DIG-labeled antisense RNA probes were generated by linearizing pTP221 (Pfirrmann et al., 2015) with *Hind*III-HF (NEB) and *in vitro* transcription with T7 RNA Polymerase (Roche) as described before (Pfirrmann et al., 2015). *Xenopus* embryos were fixed at consecutive developmental stages, and WMISH was carried out as previously described (Pfirrmann et al., 2015). Embryos probed with antisense RNAs of *pax6*, *rmnd5a* and *mkln1* were vibratome sectioned (30 μm) and photographed.

### Alcian Blue staining

Cartilage staining in *X. laevis* embryos was performed as described previously (Gessert et al., 2007). In brief; embryos were fixed in MEMFA [0.1 M MOPS (pH 7.4), 2 mM EGTA, 1 mM MgSO_4_, and 4% formaldehyde] at NF stage 45 for 2 h at room temperature. Afterward, embryos were rinsed in phosphate-buffered saline (PBS) and stained with 1% Alcian Blue containing 0.5% acetic acid for 2 h at room temperature. The staining solution was removed, and embryos were maintained in 80% ethanol and 20% acetic acid. For bleaching, embryos were first incubated for 3 h in 30% H_2_O_2_ and then 2 h in 0.05% trypsin (Gibco) in a saturated sodium tetraborate solution. After rinsing in PBST (130 mM NaCl, 7 mM Na_2_HPO_4_, 3 mM NaH_2_PO_4_, 0.1% Tween, pH 7) for several days at 4°C, the skin of the embryos was removed, and regions of interest were photographed.

### Microinjection of mRNA and visualization of cilia in *Xenopus* embryos

Embryos were injected with mRNAs at a four-cell stage using a PicoSpritzer setup in 1/3× Modified Frog Ringer's solution (MR) with 2.5% Ficoll PM 400 (GE Healthcare, 17-0300-50) (Sive et al., 2010). They were transferred after injection into 1/3× MR containing gentamicin (0.05 mg/ml). Drop size was calibrated to ∼7–8 nl per injection. mRNAs encoding Centrin4–RFP, Centrin4–CFP, Clamp–RFP (Walentek et al., 2016), and GFP–Rmnd5a were prepared using the Ambion mMessage mMachine kit (Invitrogen, AM1340) using SP6 and then diluted to ∼30–150 ng/µl (∼240–800 pg per injection) for injection into embryos. For *Xenopus* antibody staining, immunofluorescence was performed on whole-mount embryos fixed at embryonic stage 30 (multi-ciliated cells, MCCs), or stage 17 (GRP cilia) (Walentek and Quigley, 2017) in 4% paraformaldehyde at 4°C overnight. Embryos were washed 3×15 min with PBS, then 2×30 min in PBST (0.1% Triton X-100 in PBS), and were blocked in PBST-CAS [90% PBS containing 0.1% Triton X-100 and 10% CAS blocking reagent (Thermo Fisher Scientific, 00-8120)] for 1 h at room temperature. Primary mouse monoclonal anti-acetylated-tubulin antibody (1:700; Sigma-Aldrich, T6793) and secondary antibodies Alexa Fluor 555-labeled goat anti-mouse IgG antibody (1:250; Molecular Probes, A21422) or Alexa Fluor 405-labeled goat anti-mouse IgG antibody (1:250; Molecular Probes, A31553) were applied in 100% CAS blocking reagent overnight at 4°C. Actin staining was performed by incubation (∼30–60 min at room temperature) with Alexa Fluor 488-labeled phalloidin (1:40; Molecular Probes, A12379). A detailed protocol has been published previously (Walentek et al., 2014). Imaging was performed on a Zeiss LSM 700 confocal microscope using a 63× objective.

### Plasmids and oligonucleotides

Transfections were performed using Lipofectamine 2000 (Thermo Fisher Scientific) for plasmids, and Lipofectamine RNAiMAX (Thermo Fisher Scientific) for siRNAs. All other plasmids and oligonucleotides are listed in Tables S2 and S3. For knockdown experiments, siRNAs were provided by Qiagen, including negative control siRNA (Qiagen, 1022076).

### Western blotting and immunoprecipitation

Western blotting was performed as described previously (Santt et al., 2008). Cells were lysed with 50 mM Tris-HCl (pH 7.4), 2 mM EDTA, 1 mM EGTA, 50 mM NaF, 1 mM DTT, 10 mM Na_4_P_2_O_7_, 1 mM Na_3_VO_4_, 1% Triton X-100, 0.1% SDS, 0.5 mM PMSF and 1× protease inhibitor cocktail (Roche, Basel, Switzerland). Protein concentration was determined with a BCA assay (Thermo Fisher Scientific), and ∼6–20 μg was loaded per lane. Antibodies are listed in Table S4. For signal quantification, X-ray films were scanned in transparency mode and saved as 600 dpi tiff images, or images were obtained using a ChemiDoc MP Imaging System (Bio-Rad Laboratories, CA, USA) and saved as 300 dpi tiff images. Densitometry was performed with ImageJ software (NIH, Bethesda, MD, USA) using the rectangular area selection tool. Background signals were subtracted, and relative protein levels were compared with the loading control (β-actin or GAPDH).

### Subcellular protein fractionation

Cytosolic extracts were obtained using the Subcellular Protein Fractionation Kit for Cultured Cells (Thermo Fisher Scientific, 78840) according to the manufacturer's instructions. Briefly, cells were harvested then washed with ice-cold PBS. The cell pellets were suspended in ice-cold CEB buffer, incubated for 10 min at 4°C and centrifuged for 5 min at 500 ***g***. The supernatant contained the cytoplasmic extract and was used for subsequent SDS–PAGE and western blotting analysis.

### cDNA synthesis and quantitative PCR

RNA was extracted using the RNeasy Kit (Qiagen, Hilden, Germany). Human RNA used in Fig. 6D was purchased from Stratagene. Then, 1 µg RNA was reverse transcribed into cDNA in a 10 µl reaction by using HiScript II Q RT SuperMix for quantitative PCR (qPCR; Vazyme Biotech, Nanjing, China). A 20 µl qPCR reaction set contained 20 ng cDNA, 1× Maxima SYBR Green/ROX qPCR Master Mix (Thermo Fisher Scientific), 0.3 μM forward primer and 0.3 μM reverse primer. qPCRs were run on a Roche LightCycler 480 II and consisted of a uracil–DNA glycosylase pre-treatment for 2 min at 50°C, an initial hot start for 10 min at 95°C, followed by 40 cycles with a denaturation step of 15 s at 95°C and an annealing–extension step of 60 s at 60°C. Afterward, a melt curve was recorded. Each measurement was repeated three times, and each sample was analyzed in triplicate with *Hprt* (hypoxanthine phosphoribosyltransferase) as an internal control. qPCR primers are listed in Table S2. Relative expression was determined using the Abs Quant/Second Derivative Max analysis method (Horizon), is given as mean±s.e.m., and was analyzed by two-tailed unpaired Student's *t*-test.

### Immunofluorescence

Cells were grown on Millicell EZ slides (Merck Millipore, Burlington, USA) pre-treated with 0.1% gelatine solution for 30 min at 37°C, or on coverslips (Menzel, Fisher Scientific GmbH, Schwerte, Germany). Subsequently, cells were fixed with 4% paraformaldehyde or methanol for 10 min at 4°C, permeabilized, and blocked with 1× PBS containing 0.3% Triton X-100 and 3% BSA for 30 min at room temperature. Antibodies are listed in Table S4 and were used in a 1:100 dilution if not mentioned otherwise. Intensity of fluorescence was determined using ImageJ software using the freeform selection tool. Background signals were subtracted, and relative fluorescence levels were compared with the fluorescence levels of acetylated tubulin for primary cilia and DAPI for nuclei. The corrected total cell fluorescence (CTCF) was calculated using the following formula: CTCF=integrated density−(area of selected cell×mean fluorescence of background readings). The CTFC (primary cilium or nucleus) was then divided by the CTFC (acetylated tubulin or DAPI) to obtain the relative ciliary or nuclear protein level.

### Statistical analysis

Statistic values were calculated using either a two-tailed unpaired Student's *t*-test analysis or chi-square test with the GraphPad Prism 9 software. Data include values from at least three replicate experiments.

## Supplementary Material

Reviewer comments
