# Supplementary material for: Cilia-localized GID/CTLH ubiquitin ligase complex regulates protein homeostasis of sonic hedgehog signaling components
Source: J Cell Sci. 2022 May 11;135(9):jcs259209. doi: 10.1242/jcs.259209 (PMC9264362; doi:10.1242/jcs.259209)
Supplement: Reviewer comments [file joces-135-259209_review_history.pdf]

Cilia-localized GID/CTLH ubiquitin ligase complex regulates protein homeostasis of sonic hedgehog signaling components

Friederike Hantel, Huaize Liu, Lisa Fechtner, Herbert Neuhaus, Jie Ding, Danilo Arlt, Peter Walentek, Pablo Villavicencio-Lorini, Christoph Gerhardt, Thomas Hollemann and Thorsten Pfirrmann
DOI: 10.1242/jcs.259209

Editor: John Heath

Review timeline
Original submission: 2 August 2021
Editorial decision: 14 October 2021
First revision received: 20 February 2022
Accepted: 24 March 2022

Original submission

First decision letter

MS ID#: JOCES/2021/259209

MS TITLE: Cilia-localized GID/CTLH ubiquitin ligase complex regulates protein homeostasis of Sonic Hedgehog signaling components and knockdown produces ciliopathy-like phenotypes in vivo

AUTHORS: Friederike Hantel, Huaize Liu, Lisa Fechtner, Jie Ding, Danilo Arlt, Peter Walentek, Pablo Villavicencio-Lorini, Christoph Gerhardt, Thomas Hollemann, and Thorsten Pfirrmann
ARTICLE TYPE: Research Article

We have now reached a decision on the above manuscript.

To see the reviewers' reports and a copy of this decision letter, please go to: https://submit-jcs.biologists.org and click on the 'Manuscripts with Decisions' queue in the Author Area. (Corresponding author only has access to reviews.)

As you will see, the reviewers raise a number of criticisms that prevent me from accepting the paper at this stage. They suggest, however, that a revised version might prove acceptable, if you can address their concerns. If you think that you can deal satisfactorily with the criticisms on revision, I would be pleased to see a revised manuscript. We would then return it to the reviewers.

We are aware that you may be experiencing disruption to the normal running of your lab that makes experimental revisions challenging. If it would be helpful, we encourage you to contact us to discuss your revision in greater detail. Please send us a point-by-point response indicating where you are able to address concerns raised (either experimentally or by changes to the text) and where you will not be able to do so within the normal timeframe of a revision. We will then provide further guidance. Please also note that we are happy to extend revision timeframes as necessary.

Please ensure that you clearly highlight all changes made in the revised manuscript. Please avoid using 'Tracked changes' in Word files as these are lost in PDF conversion.

© 2022. Published by The Company of Biologists under the terms of the Creative Commons Attribution License (https://creativecommons.org/licenses/by/4.0/).

I should be grateful if you would also provide a point-by-point response detailing how you have dealt with the points raised by the reviewers in the 'Response to Reviewers' box. Please attend to all of the reviewers' comments. If you do not agree with any of their criticisms or suggestions please explain clearly why this is so.

Reviewer 1

Advance summary and potential significance to field

The manuscript by Hantel et al postulates the role of a cilia-localized GID/CTLH ubiquitin ligase complex in protein homeostasis of Hedgehog (Hh) signaling components. They also suggest that knockdown of these components in Xenopus produces ciliopathy-like phenotypes in vivo. Overall, the manuscript would benefit from additional control experiments and reevaluation of the data on some of the Hh signaling phenotypes as described below.

Comments for the author

Major comments:
1. The authors nicely show the localization of GID complex proteins in basal body (TWA1 and RMND5A) or inside the cilium (MKLN1 and ARMC8). However, key controls, such as testing in knockdown/knockout cells, for the antibodies are missing.
2. Levels of Gli2 in cilia are affected in RMND5A knockout cells. However, as per literature, Gli2 is localized to primarily in ciliiary tips predominantly upon Hh pathway activation. Instead, the authors see Gli2 localized throughout cilia and to be unaffected upon SAG treatment in control cells.
3. Hh phenotypes seen in RMND5A knockout cells for the postulated Hh pathway proteostasis phenotypes are subtle and need to tested for rescue upon add back of RMND5A. Other subunits in the GID complex could be tested by knockdown, if possible.
4. The authors suggest that knockdown of Rmd5a in Xenopus produces ciliopathy-like phenotypes with overlap in MKS and OPD syndromes. I am not sure if the current phenotypic analyses suggest such overlap.

Minor.
1. Sources of GID complex antibodies have not been mentioned.
2. Levels of Gli1 transcript upregulation in WT cells upon SAG treatment is very different between panels in Figures 3-4.
3. Skeletal staining phenotypes in Fig 6G needs to be highlighted for the general reader.
4. Although GID functions as a complex, the authors show that the different components localized to either basal body or ciliary axoneme. Please discus if they can function independently.

Reviewer 2

Advance summary and potential significance to field

In the manuscript entitled “Cilia-localized GID/CTLH ubiquitin ligase complex regulates protein homeostasis of Sonic Hedgehog signaling components and knockdown produces ciliopathy-like phenotypes in vivo” from Hantel and colleagues. the authors show that components of the GID/CTLH complex, known to regulate AMPK activity, localize to the ciliary basal body (or axoneme) and that a member of this complex, RMND5A, is required for induction of the Hedgehog (Hh) signaling pathway. Such regulation of Hh signaling by RMND5A appears to be independent of its role in AMPK and the mTOR regulation and the data suggest that RMND5A functions by promoting the efficient ciliary localization of specific Hh signal transduction machinery (Gli2 and Patched1). The authors further show that expression of RNAs encoding components of the GID/CTLH complex is found in ciliated tissues in the Xenopus embryo and morpholino-mediated knockdown of rmnd5 in Xenopus results in defects consistent with defective ciliary function. Collectively, the work represents an interesting and potentially important advance in the field of ciliar signaling as the mechanisms that control the ciliary import/homeostasis of signal transduction components are not well understood.

© 2022. Published by The Company of Biologists under the terms of the Creative Commons Attribution License (https://creativecommons.org/licenses/by/4.0/).

Comments for the author

The work from Hantel et al., has generally been well executed and it supports arguments and conclusions made by the authors. However, there is one area where further experiments are warranted, a point of confusion requiring clarification, and a minor issue with wording:

a) The authors prevent evidence that knockdown of rmnd5 in Xenopus results in abnormalities of the brain, eye, and craniofacial cartilage. These phenotypes may result from defects in ciliary function and/or Hedgehog signaling but further investigation of the in vivo model to tie this back to the in vitro work should be done. Specifically, the authors should at least anlayze cilia in the morphants (length, frequency and including, if possible, the localization of cilia proteins). More importantly, to address an in vivo role for rmnd5 with respect to Hh signaling, the authors should analyze readouts of the Hh pathway in the morhpants (e.g., Gli1 — assayed by in situ hybridization or qPCR). In addition, co-injection of a construct overexpressing Shh along with the rmnd5, (in parallel with the Shh construct on its own) would provide a more sensitized system to test whether loss of rmnd5 can rescue the phenotypes of Shh overexpression and therefore participates in xenopus Hh signaling.

b) In figure 4F, the authors show that treatment of cells with Compound C (dorsomorphin) suppresses the effects of SAG treatment on Gli1 expression. This seems odd because, as I understand it, Compound C is an antagonist of AMPK activity and AMPK antagonizes Hh pathway activity by phosphorylating and destabilizing Gli1. First, the authors should explain better what Compound C is (it seems to come out of nowhere in the paper). Second, and more importantly, that authors should clarify why Compound C would seem to antagonize Hh pathway activity here since Compound C should inhibit an inhibitor of the Hh pathway (AMPK) – I would have predicted stronger, not weaker, Hh responses. Am I missing something here?

c) Lastly (a minor point): on page 6, the authors write, : “This suggests a function of the cilia-localized GID-complex in the regulation of ciliary SHH-protein homeostasis.” This statement is confusing and should be reworded as it suggests the GID complex controls homeostasis of the SHH ligand (rather—it should be regarding Hh signal transducing proteins).

First revision

<u>Author response to reviewers' comments</u>

Dear Reviewers,
We appreciate your valuable and helpful comments on our manuscript entitled “Cilia-localized GID/CTLH ubiquitin ligase complex regulates protein homeostasis of Sonic Hedgehog signaling components” (Manuscript JOCES/2021/259209) and have taken these comments as a guide for the correction and quality improvement of our manuscript. Every point you made was carefully considered and revised. Your questions/comments are written in italic letters, our answers not.
The following points were made:

Reviewer #1:

Reviewer 1 Advance Summary and Potential Significance to Field: The manuscript by Hantel et al postulates the role of a cilia-localized GID/CTLH ubiquitin ligase complex in protein homeostasis of Hedgehog (Hh) signalling components. They also suggest that knockdown of these components in Xenopus produces ciliopathy-like phenotypes in vivo. Overall, the manuscript would benefit from additional control experiments and reevaluation of the data on some of the Hh signaling phenotypes as described below.

Reviewer 1 Comments for the Author:

Major comments:

1. The authors nicely show the localization of GID complex proteins in basal body (TWA1 and RMND5A) or inside the cilium (MKLN1 and ARMC8). However, key controls, such as testing in knockdown/knockout cells, for the antibodies are missing.

© 2022. Published by The Company of Biologists under the terms of the Creative Commons Attribution License (https://creativecommons.org/licenses/by/4.0/).

We thank reviewer #1 for appreciating the fact that GID complex subunits co-localize with basal bodies and the ciliary axoneme. Antibodies used in this study were previously tested for specificity by the manufacturer and in several publications. We particularly chose these antibodies (anti-TWA1, anti-ARMC8, anti-MKLN1), because no/little cross-reactivity can be observed in Western analysis and bands are reduced upon knockdown of corresponding proteins (Maitland et al., 2019). In case of the anti-RMND5A antibody, we used NIH 3T3 cells lacking RMND5A (KO NIH3T3) in order to test antibody specificity (see images below) and added Supplementary Figure 4 to the manuscript. To make this clear we added the sentence 'Specificities of the used antibodies were either tested by Western blot analysis for anti- RMND5A (**Figure S4**) or in previous publications for anti-TWA1, anti-ARMC8, anti-MKLN1 (Maitland et al., 2019).' to the manuscript (p.7). Furthermore, we skipped primary antibodies to control for the possibility of signal overlap in the different channels and used overexpression of RMND5A-GFP constructs in cell lines and in vivo to exclude unspecific localization at the basal body which is not the case in GFP expressing constructs and in basal bodies of multi- ciliated cells of the skin but at centrioles of adjacent cell types (Supplementary Figure 2).

[***NOTE***: *We have removed unpublished data that had been provided for the referees in confidence.*]

2. Levels of Gli2 in cilia are affected in RMND5A knockout cells. However, as per literature, Gli2 is localized to primarily in ciliiary tips predominantly upon Hh pathway activation. Instead, the authors see Gli2 localized throughout cilia and to be unaffected upon SAG treatment in control cells.

Reviewer #1 raised a critical point. We added to the manuscript 'In 2005, Haycraft and colleagues showed that the GLI proteins (GLI1, GLI2 and GLI3) localize at the distal tips of primary cilia without any stimulation of HH signalling (Haycraft et al., 2005). From then on, a lot of studies revealed an accumulation of the GLI proteins at the ciliary tip upon HH signaling stimulation (Chen et al., 2009; Massa et al., 2019; Moon et al., 2014; Wen et al., 2010; Yang et al., 2015; Yoshida et al., 2020). However, several reports described a presence of GLI2 and GLI3 in the entire cilium without HH stimulation (Clement et al., 2009; Egeberg et al., 2012; Emechebe et al., 2016; Kiprilov et al., 2008) reflecting the transport of the GLI proteins from the ciliary base to the distal tip and vice versa (Eguether et al., 2014; Kim et al., 2009; Qin et al., 2011). In this context, it was demonstrated that the localization of GLI3 along the entire cilium is not changed by SAG treatment (Emechebe et al., 2016). In our study, SAG treatment does not alter the ciliary localization of GLI2 but – importantly – leads to a consistent and significant upregulation of target gene expression demonstrating robust pathway activation upon SAG treatment.' on page 14.

3. Hh phenotypes seen in RMND5A knockout cells for the postulated Hh pathway proteostasis phenotypes are subtle and need to tested for rescue upon add back of RMND5A. Other subunits in the GID complex could be tested by knockdown, if possible.

We have taken this comment very seriously and performed rescue experiments with RMND5A overexpressing plasmids in the KO context. In our experiments overexpression of RMND5A is not sufficient to complement for SHH signalling defects (see below, Figure 1). At first, this result seemed counterintuitive and suggested off-target effects, however we performed several experiments which suggest that overexpression of GID-components causes defects in ciliogenesis and cilia function and thus interfere with SHH signalling as well.

Nonetheless, we consider the SHH-and cilium-phenotype specific to RMND5A for the following reasons: (1) RMND5A knock out results in significant elongation of primary cilia, a phenotype that can be phenocopied by siRNA induced knock-down of RMND5A (Liu et al., 2020). (2) We have tested a second independent KO clone that we retrieved from CRISPR- Cas9 mutation and tested SAG induced Sonic Hedgehog signalling. Our second clone (clone 33) shows an almost complete lack of SAG induced GLI1 induction and behaves comparable to clone 32 (see below, Figure 3). (3) siRNA knock down phenocopies the SHH signalling defect (see Figure 2

© 2022. Published by The Company of Biologists under the terms of the Creative Commons Attribution License (https://creativecommons.org/licenses/by/4.0/).

manuscript). (4) GID-subunits localize to the primary cilium (see Figure 1 manuscript) and (5) Phenotypes in Xenopus can be traced back to defects in SHH signaling (see Figure 7, manuscript).

Furthermore, we performed additional overexpression experiments with GID-complex components that might explain the non-rescue phenotype. We generated a new plasmid expressing a GID4-GFP fusion protein and transfected cells with either a control GFP plasmid or the GID4-GFP expressing plasmid. Thereafter, we induced cilia and counted green cells with cilia and without cilia. The graph below shows that overexpression of GID4 results in significant reduction of cells with primary cilia when compared to cells transfected with a GFP expressing plasmid (Figure 4, below). Similarly, the overexpression of RMND5A-GFP affected ciliogenesis (Figure 2, see below). We reason that a carefully balanced level of GID-subunits is critical for ciliogenesis and cilia function and conclude that overexpression of GID-subunits can interfere with SHH signalling and thus results in the non-rescue. At this time we do not fully understand the molecular reason for this result and thus wish to exclude such data from this manuscript.

[***NOTE:*** *We have removed unpublished data that had been provided for the referees in confidence.*]

[***NOTE:*** *We have removed unpublished data that had been provided for the referees in confidence.*]

4. The authors suggest that knockdown of Rmd5a in Xenopus produces ciliopathy-like phenotypes with overlap in MKS and OPD syndromes. I am not sure if the current phenotypic analyses suggest such overlap.

We agree with reviewer #1. Phenotypic comparison between species is fundamentally associated with limitations. References to certain syndromal diseases in humans can only be traced in rough patterns of organ involvement. However, at the outset of this study, the observed morpholino-based knockdown phenotypes in Xenopus and the work of Vogel et al. describing an association between the RMND5A locus and occipital encephalocele were the focus and guided us to the connection with cilia (Vogel et al., 2012). In particular, the cartilage phenotype overlaps with phenotypes described in the context of components of SHH signaling; see: http://www.xenbase.org/common/showPhenotype.do?method=display&phenoId=23769.

To make this clear to the reader we added a section in the discussion of the manuscript to highlight that direct comparisons of phenotypes between species is associated with limitations and furthermore deleted reference to MKS from the manuscript on p.6 l.19 we deleted 'with a Meckel-Gruber-like syndrome'.

To provide further evidence of ciliopathy-like phenotypes we performed additional experiments in Xenopus laevis that largely support our hypothesis that knockdown of rmnd5 results in defects in ciliary function and SHH-signaling (see Figure 7). We followed spatiotemporal expression of *sox2* (pan-neural marker), *shh* (axial mesoderm and *shh* target gene in the floorplate) and *nkx2.1* (marker of the ventral forebrain) by whole-mount in situ hybridization (Figure 7). From the novel data we conclude that *rmnd5* function doesn't interfere with primary neural induction in the neural plate territory as monitored by sox2 expression at NF stage 14 as well as with the expression domains of *shh* that appears almost normal. Our new data suggests, that induction of the neural plate territory as well as production of SHH morphogen seems mostly unaffected. The development of the forebrain and eye appears hindered as the head of the injected side is smaller, evident by the shift of the olfactory anlage, missing stratification of the eye and smaller eyes including a missing lens. Transcripts of *nkx2.1,* a marker of the ventral forebrain, were additionally monitored to illustrate patterning events of the prospective forebrain, which highly depend on *shh* signaling. As result we show that the expression of *nkx2.1* is clearly reduced on the injected side (Figure 7) supporting our hypothesis that RMND5A/GID2 depletion interferes with SHH signal transduction and likely SHH patterning.

© 2022. Published by The Company of Biologists under the terms of the Creative Commons Attribution License (https://creativecommons.org/licenses/by/4.0/).

Minor comments:

1. Sources of GID complex antibodies have not been mentioned.

We thank the reviewer for this very important point, we have included the missing antibodies in the antibody list in the manuscript on page 25. Additionally, we have included the sentence ‘Additional antibodies were diluted 1:100 in PBS containing 3% BSA and 0,3% Tween-20 and used to visualize subunits of the GID-complex (TWA1, RMND5A, ARMC8, MKLN1).’ in the Figure legends on page 26.

2. Levels of Gli1 transcript upregulation in WT cells upon SAG treatment is very different between panels in Figures 3-4.

We agree with reviewer #1. Importantly, the differences mentioned by reviewer #1 can also be seen in *in vitro* experiments of several previous studies (Diao et al., 2018; Eguether et al., 2018; Hasegawa et al., 2017) suggesting that the conditions of *in vitro* experiments might cause these differences in the expression of *Gli1*. However, we find consistent and statistical significant upregulation of *Gli1* transcripts upon SAG treatment in all of our experiments ensuring the reliability of our data.

3. Skeletal staining phenotypes in Fig 6G needs to be highlighted for the general reader.

We have described the cartilage staining phenotypes and its relation to SHH-signaling and ciliopathies for the general reader and added the sentences ‘Craniofacial development is initiated by cranial neural crest cells that migrate from the dorsal neural tube into a series of branchial arches to give rise to the Meckel’s and palatoquadrate cartilages, the palatoquadrate cartilages and the branchial cartilages (Baltzinger et al., 2005) (Figure 6G, b). Craniofacial defects can be visualized by Alcian blue staining in the developing tadpole (Abramyan 2019).’ We have included the text in the manuscript at p.15 line18.

4. Although GID functions as a complex, the authors show that the different components localized to either basal body or ciliary axoneme. Please discus if they can function independently.

We are also intrigued by the differing localization of different GID-subunits. Recent data from the yeast GID-complex show that it is existing with various subunit compositions. Also the human GID-complex seems to appear with e.g. either MKLN1 or WDR26 (Lampert et al., 2018). Consequently, it is possible that different localisations can be explained by different subunit compositions or by functions independent of the complex. Interestingly the ciliary proteasome behaves very similar and subunits can be found at the basal body, others at the axoneme (Gerhardt et al., 2015). To make this clear to the reader we have added the sentence ‘At this stage it is unknown whether the differing distribution of GID-complex subunits can be explained by a function independent of the GID-complex or by different subunit compositions that localize either to the basal body or the axoneme.’ in the discussion.

Reviewer #2:

Reviewer 2 Advance Summary and Potential Significance to Field: In the manuscript entitled “Cilia-localized GID/CTLH ubiquitin ligase complex regulates protein homeostasis of Sonic Hedgehog signaling components and knockdown produces ciliopathy-like phenotypes in vivo” from Hantel and colleagues. the authors show that components of the GID/CTLH complex, known to regulate AMPK activity, localize to the ciliary basal body (or axoneme) and that a member of this complex, RMND5A, is required for induction of the Hedgehog (Hh) signaling pathway. Such regulation of Hh signaling by RMND5A appears to be independent of its role in AMPK and the mTOR regulation and the data suggest that RMND5A functions by promoting the efficient ciliary localization of specific Hh signal transduction machinery (Gli2 and Patched1). The authors further show that expression of RNAs encoding components of the GID/CTLH complex is found in ciliated tissues in the Xenopus embryo and morpholino-mediated knockdown of rmnd5 in Xenopus results in defects consistent with defective ciliary function. Collectively, the work represents an

© 2022. Published by The Company of Biologists under the terms of the Creative Commons Attribution License (https://creativecommons.org/licenses/by/4.0/).

interesting and potentially important advance in the field of ciliar signaling as the mechanisms that control the ciliary import/homeostasis of signal transduction components are not well understood.

Reviewer 2 Comments for the Author: The work from Hantel et al., has generally been well executed and it supports arguments and conclusions made by the authors. However, there is one area where further experiments are warranted, a point of confusion requiring clarification, and a minor issue with wording:

a) The authors prevent evidence that knockdown of rmnd5 in Xenopus results in abnormalities of the brain, eye, and craniofacial cartilage. These phenotypes may result from defects in ciliary function and/or Hedgehog signaling but further investigation of the in vivo model to tie this back to the in vitro work should be done. Specifically, the authors should at least anlayze cilia in the morphants (length, frequency and including, if possible, the localization of cilia proteins).

We thank reviewer #2 for this comment. To provide further evidence that knockdown of *rmnd5* in Xenopus results in defects in ciliary function and SHH signaling, we expanded our phenotypic studies. To knock-down *rmnd5* translation, antisense morpholino together with synthetic RNA encoding nuclear *beta-galactosidase* were injected into one cell of 2-cell stage embryos to mark the injected side of the larvae visible as red nuclear staining. We followed spatiotemporal expression of *sox2* (pan-neural marker), *shh* (axial mesoderm and *shh* target gene in the floorplate) and *nkx2.1* (marker of the ventral forebrain) by whole-mount in situ hybridization (Figure 7). We can conclude from the data that *rmnd5* function doesn't interfere with primary neural induction in the neural plate territory as monitored by *sox2* expression at NF stage 14 as well as with the expression domains of *shh* that appeared almost normal (Figure 7A and B). The data suggest that induction of the neural plate territory as well as production of SHH morphogen seems mostly unaffected. To test whether SHH signal transduction is impaired, the expression of the SHH target gene *nkx2.1,* a marker of the ventral forebrain, was monitored. Remarkably, the expression of *nkx2.1* is clearly reduced on the injected side (Figure 7C) revealing disturbed SHH signaling in *rmnd5a*-deficient Xenopus forebrains. Interestingly, SHH signals from the notochord are able to induce the SHH expression in the floor plate in the absence of RMND5A although SHH signaling is impeded. A similar situation has been reported in the neural tube of *Ft/Ft* mouse embryos at E10.5 in which the induction of *Shh* expression in the floor plate was successful but SHH target expression in the ventral neural tube was hampered (Gotz et al., 2005). In these embryos, six genes – *Irx3*, *Irx5*, *Irx6*, *Fto*, *Fts* and *Rpgrip1l* – are deleted (Peters et al., 2002). One of the products of these genes, Rpgrip1l, localizes to the ciliary transition zone and is involved in SHH signal transduction (Gerhardt et al., 2015; Vierkotten et al., 2007; Wiegering et al., 2019). Importantly, morphogenesis of the forebrain and the eyes is strongly perturbed in *Ft/Ft* mouse embryos (Anselme et al., 2007) and resembles the phenotype of the *rmnd5a*-deficient side in *Xenopus*. We reason that the forebrain and eye phenotype of *Ft/Ft* embryos might be caused by the loss of Rpgrip1l, since *Rpgrip1l*- negative mouse embryos display defects in forebrain and eye development as well (Besse et al., 2011; Delous et al., 2007; Vierkotten et al., 2007). Remarkably, mutations in *RPGRIP1L* result in severe human ciliopathies (Delous et al., 2007). All these findings point to a close relationship between the GID-complex, SHH signaling, cilia and ciliopathies. Concerning our *in vitro* and *in vivo* data on SHH signaling, we hypothesize that the lack of target gene activation in the absence of RMND5A is a result of the reduced amount of SHH signaling components in the cilium.

Corresponding sections were added to the manuscript on page 14 and highlighted in red.

More importantly, to address an in vivo role for rmnd5 with respect to Hh signaling, the authors should analyze readouts of the Hh pathway in the morhpants (e.g., Gli1 — assayed by in situ hybridization or qPCR).

As outlined above, we analyzed the expression of the HH target *nkx2.1* in forebrain development of Xenopus after the knock-down of *rmnd5a.* The observed reduced expression of *nkx2.1* reflects an impaired SHH signaling in the absence of Rmnd5a.

In addition, co-injection of a construct overexpressing Shh along with the rmnd5, (in parallel with the Shh construct on its own) would provide a more sensitized system to test whether loss

© 2022. Published by The Company of Biologists under the terms of the Creative Commons Attribution License (https://creativecommons.org/licenses/by/4.0/).

of rmnd5 can rescue the phenotypes of Shh overexpression and therefore participates in xenopus Hh signaling.

The Sonic hedgehog morphogen acts in a graded manner to pattern e.g. the ventral neural tube. The balanced distribution of this morphogen in a gradient is critical for development. Changing this gradient by overexpressing Shh will cause severe developmental phenotypes, most likely being lethal (https://doi.org/10.1038/emboj.2009.12). Our experiments consistently show that SHH signalling is affected upon reduction of GID-complex activity by qPCR as well as patterning of the forebrain as detected by *nkx2.1* whole mount in situ and other experiments.

b) In figure 4F, the authors show that treatment of cells with Compound C (dorsomorphin) suppresses the effects of SAG treatment on Gli1 expression. This seems odd because, as I understand it, Compound C is an antagonist of AMPK activity and AMPK antagonizes Hh pathway activity by phosphorylating and destabilizing Gli1. First, the authors should explain better what Compound C is (it seems to come out of nowhere in the paper).
We thank referee #2 for this comment. To make it clear to the reader what Compound C really is, we added the sentence 'Dorsomorphin (Compound C) is an established selective inhibitor of AMPK displaying no significant inhibition of other kinases and consequently influences AMPK-dependent signaling pathways (Zhou et al., 2001)' on p.8 highlighted in red.

Second, and more importantly, that authors should clarify why Compound C would seem to antagonize Hh pathway activity here since Compound C should inhibit an inhibitor of the Hh pathway (AMPK) – I would have predicted stronger, not weaker, Hh responses. Am I missing something here?

We thank Referee #2 for pointing that out. Compound C inhibits AMPK signaling. Since AMPK signaling negatively regulates HH signaling, an upregulation of HH signaling by Compound C seems to be obvious. We administered the AMPK inhibitor Compound C to SAG- treated wild type cells and detected a decreased SHH target gene expression (Figure 4F) indicating that AMPK positively regulates HH signaling in NIH-3T3 cells. Since the negative regulatory effect of AMPK on SHH signaling was shown in NIH-3T3 cells (Di Magno et al., 2016; Li et al., 2015), it is unlikely that AMPK has a cell type-specific effect on SHH signaling. However, Compound C has also an inhibitory effect on PDGFRß. It was demonstrated that this inhibitory effect is independent of AMPK signaling (Kwon et al., 2013) and that PDGFRß signaling activates HH signaling resulting in an increased expression of the HH target gene *Gli1* (Fingas et al., 2011). Thus, it is conceivable that the reduced HH signaling caused by treatment with Compound C in our work could be traced back to a side effect of Compound C such as its inhibitory effect on PDGFRß. We inserted this possibility in the discussion part of the revised manuscript p15 line 8: 'Since the negative regulatory effect of AMPK on SHH signaling was shown in NIH-3T3 cells (Di Magno et al., 2016; Li et al., 2015), it is unlikely that AMPK has a cell type-specific effect on SHH signaling. Potentially, the observed reduction of HH target gene expression caused by treatment with Compound C could be traced back to a side effect of Compound C. Kwon et al. described an inhibitory and AMPK-independent effect of Compound C on platelet-derived growth factor receptor ß (PDGFRß) (Kwon et al., 2013). It is already known that PDGFRß signaling activates SHH signaling resulting in an increased expression of the SHH target gene *Gli1* (Fingas et al., 2011). Accordingly, the reduced SHH target gene expression in combined-treated (SAG and Compound C) wild type NIH-3T3 cells could be triggered by the inhibition of PDGFRß.'

A section in the discussion was added on p.14-15 to highlight this.

c) Lastly (a minor point): on page 6, the authors write, : "This suggests a function of the cilia-localized GID-complex in the regulation of ciliary SHH-protein homeostasis." This statement is confusing and should be reworded as it suggests the GID complex controls homeostasis of the SHH ligand (rather—it should be regarding Hh signal transducing proteins).

We thank Referee 2 for this comment. In order to minimize confusion, we rephrased the sentence and wrote 'This suggests a function of the cilia-localized GID-complex in regulating the proteostasis of several SHH signaling components within the primary cilium.' instead.

© 2022. Published by The Company of Biologists under the terms of the Creative Commons Attribution License (https://creativecommons.org/licenses/by/4.0/).

References:

Chen, M., Wilson, C., Li, Y., Law, K., Lu, C., Gacayan, R., Zhang, X., Hui, C. and Chuang, P. (2009). Cilium-independent regulation of Gli protein function by Sufu in Hedgehog signaling is evolutionarily conserved. *Genes Dev.* **23**, 1910-1928.

Clement, C., Kristensen, S., Møllgård, K., Pazour, G., Yoder, B., Larsen, L. and Christensen, S. (2009). The primary cilium coordinates early cardiogenesis and hedgehog signaling in cardiomyocyte differentiation. *J. Cell Sci.* **122**, 3070-3082.

Di Magno, L., Basile, A., Coni, S., Manni, S., Sdruscia, G., D'Amico, D., Antonucci, L., Infante, P., De Smaele, E., Cucchi, D. et al. (2016). The energy sensor AMPK regulates Hedgehog signaling in human cells through a unique Gli1 metabolic checkpoint. *Oncotarget* **7**, 9538-9549.

Diao, Y., Rahman, M., Vyatkin, Y., Azatyan, A., St Laurent, G., Kapranov, P. and Zaphiropoulos, P. (2018). Identification of novel GLI1 target genes and regulatory circuits in human cancer cells. *Mol. Oncol.* **12**, 1718-1734.

Egeberg, D., Lethan, M., Manguso, R., Schneider, L., Awan, A., Jørgensen, T., Byskov, A., Pedersen, L. and Christensen, S. (2012). Primary cilia and aberrant cell signaling in epithelial ovarian cancer. *Cilia* **1**, 15.

Eguether, T., Cordelieres, F. and Pazour, G. (2018). Intraflagellar transport is deeply integrated in hedgehog signaling. *Mol. Biol. Cell* **29**.

Eguether, T., San Agustin, J., Keady, B., Jonassen, J., Liang, Y., Francis, R., Tobita, K., Johnson, C., Abdelhamed, Z., Lo, C. et al. (2014). IFT27 links the BBSome to IFT for maintenance of the ciliary signaling compartment. *Dev. Cell* **31**, 279-290.

Emechebe, U., Kumar, P. P., Rozenberg, J., Moore, B., Firment, A., Mirshahi, T. and Moon, A. (2016). T-box3 is a ciliary protein and regulates stability of the Gli3 transcription factor to control digit number. *Elife* **5**, pii: e07897.

Fingas, C., Bronk, S., Werneburg, N., Mott, J., Guicciardi, M., Cazanave, S., Mertens, J., Sirica, A. and Gores, G. (2011). Myofibroblast-derived PDGF-BB promotes Hedgehog survival signaling in cholangiocarcinoma cells. *Hepatology* **54**, 2076-2088.

Gerhardt, C., Lier, J., Burmühl, S., Struchtrup, A., Deutschmann, K., Vetter, M., Leu, T., Reeg, S., Grune, T. and Rüther, U. (2015). The transition zone protein Rpgrip1l regulates proteasomal activity at the primary cilium. *J. Cell Biol.* **210**, 115-133.

Hasegawa, D., Ochiai-Shino, H., Onodera, S., Nakamura, T., Saito, A., Onda, T., Watanabe, K., Nishimura, K., Ohtaka, M., Nakanishi, M. et al. (2017). Gorlin syndrome-derived induced pluripotent stem cells are hypersensitive to hedgehog-mediated osteogenic induction. *PLoS One* **12**, e0186879.

Haycraft, C., Banizs, B., Aydin-Son, Y., Zhang, Q., Michaud, E. and Yoder, B. (2005). Gli2 and Gli3 localize to cilia and require the intraflagellar transport protein polaris for processing and function. *PLoS Genet.* **1**, e53.

Kim, J., Kato, M. and Beachy, P. (2009). Gli2 trafficking links Hedgehog-dependent activation of Smoothened in the primary cilium to transcriptional activation in the nucleus. *Proc. Natl. Acad. Sci. U S A.* **106**, 21666-71.

Kiprilov, E., Awan, A., Desprat, R., Velho, M., Clement, C., Byskov, A., Andersen, C., Satir, P., Bouhassira, E., Christensen, S. et al. (2008). Human embryonic stem cells in culture possess primary cilia with hedgehog signaling machinery. *J. Cell Biol.* **180**, 897-904.

Kwon, H., Kim, G., Lee, Y., Jeong, M., Kang, I., Yang, D. and Yeo, E. (2013). Inhibition of platelet- derived growth factor receptor tyrosine kinase and downstream signaling pathways by Compound C. *Cell. Signal.* **25**, 883-897.

Lampert, F., Stafa, D., Goga, A., Soste, M., Gilberto, S., Olieric, N., Picotti, P., Stoffel, M. and Peter, M. (2018). The multi-subunit GID/CTLH E3 ubiquitin ligase promotes cell proliferation and targets the transcription factor Hbp1 for degradation. *Elife* **7**, e35528.

Li, Y.-H., Luo, J., Mosley, Y.-Y. C., Hedrick, V. E., Paul, L. N., Chang, J., Zhang, G., Wang, Y.-K., Banko, M. R., Brunet, A. et al. (2015). AMP-Activated Protein Kinase Directly Phosphorylates and Destabilizes Hedgehog Pathway Transcription Factor GLI1 in Medulloblastoma. *Cell reports* **12**, 599-609.

Maitland, M., Onea, G., Chiasson, C., Wang, X., Ma, J., Moor, S., Barber, K., Lajoie, G., Shaw, G. and Schild-Poulter, C. (2019). The mammalian CTLH complex is an E3 ubiquitin ligase that targets its subunit muskelin for degradation. *Sci. Rep.* **9**, 9864.

Massa, F., Tammaro, R., Prado, M., Cesana, M., Lee, B., Finley, D., Franco, B. and Morleo, M. (2019). The deubiquitinating enzyme Usp14 controls ciliogenesis and Hedgehog signaling. *Hum. Mol. Genet.* **28**, 764-777.

© 2022. Published by The Company of Biologists under the terms of the Creative Commons Attribution License (https://creativecommons.org/licenses/by/4.0/).

Moon, H., Song, J., Shin, J., Lee, H., Kim, H., Eggenschwiller, J., Bok, J. and Ko, H. (2014). Intestinal cell kinase, a protein associated with endocrine-cerebro-osteodysplasia syndrome, is a key regulator of cilia length and Hedgehog signaling. *Proc. Natl. Acad. Sci. U S A.* **111**, 8541-8546.

Qin, J., Lin, Y., Norman, R., Ko, H. and Eggenschwiler, J. (2011). Intraflagellar transport protein 122 antagonizes Sonic Hedgehog signaling and controls ciliary localization of pathway components. *Proc. Natl. Acad. Sci. U S A* **108**, 1456-1461.

Vogel, T., Manjila, S. and Cohen, A. (2012). Novel neurodevelopmental disorder in the case of a giant occipitoparietal meningoencephalocele. *Neurosurg. Pediatr.* **10**, 25-29.

Wen, X., Lai, C., Evangelista, M., Hongo, J., de Sauvage, F. and Scales, S. (2010). Kinetics of hedgehog-dependent full-length Gli3 accumulation in primary cilia and subsequent degradation. *Mol. Cell Biol.* **30**, 1910-1922.

Yang, N., Li, L., Eguether, T., Sundberg, J., Pazour, G. and Chen, J. (2015). Intraflagellar transport 27 is essential for hedgehog signaling but dispensable for ciliogenesis during hair follicle morphogenesis. *Development* **142**, 2194-2202.

Yoshida, S., Aoki, K., Fujiwara, K., Nakakura, T., Kawamura, A., Yamada, K., Ono, M., Yogosawa, S. and Yoshida, K. (2020). The novel ciliogenesis regulator DYRK2 governs Hedgehog signaling during mouse embryogenesis. *Elife* **9**, e57381.

Second decision letter

MS ID#: JOCES/2021/259209

MS TITLE: Cilia-localized GID/CTLH ubiquitin ligase complex regulates protein homeostasis of Sonic Hedgehog signaling components

AUTHORS: Friederike Hantel, Huaize Liu, Herbert Neuhaus, Lisa Fechtner, Jie Ding, Danilo Arlt, Peter Walentek, Pablo Villavicencio-Lorini, Christoph Gerhardt, Thomas Hollemann, and Thorsten Pfirrmann
ARTICLE TYPE: Research Article

I am happy to tell you that your manuscript has been accepted for publication in Journal of Cell Science, pending standard ethics checks.

Reviewer 1

Advance summary and potential significance to field

The authors have made significant revisions to the original manuscript.

Comments for the author

There are certain sections that need further attention.
1. For antibody controls, the authors show an immunoblot using RMND5A ko. They can use the same line to show specificity of the antibody used in immunofluorescence in Fig 2.
2. The data in Figure 5 shows Gli2 staining throughout cilia that is not complying with the published literature (eg please see Kim 2009 PNAS, cited twice as Kim 2009a/b in the manuscript).
3. The new data using morpholino in Fig 7 needs to be shown with appropriate controls. For eg, was the control side also injected with control oligos especially as the injected side neural tube shows "slightly retarded development"? The Nkx2.2 RNA ISH data is interesting and should be quantified with respect to control side and number of embryos tested should be mentioned.

© 2022. Published by The Company of Biologists under the terms of the Creative Commons Attribution License (https://creativecommons.org/licenses/by/4.0/).

Reviewer 2

Advance summary and potential significance to field

First, please accept my apologies for the long delay in reviewing the revised manuscript, “Cilia-localized GID/CTLH ubiquitin ligase complex regulates protein homeostasis of Sonic Hedgehog signaling components” (JOCES/2021/259209) by Hantel and colleagues. The authors have satisfactorily addressed my concerns with the original submission.
Specifically, the in situ hybridization data inn figure 7 indeed indicate that rmnd5a knockdown results in a reduction of the nkx2.1 expression domain in the brain on the injected side, which supports the conclusion that rmnd5a is required for proper Hh signal transduction. This effect does not appear to stem from changes in shh expression. I also appreciate the explanation for the unexpected effect of Compound C on Hh target gene expression, given the data from other groups indicating that Compound C inhibits PDGFRβ, and that PDGFRβ potentiates Hh signaling. Finally, I appreciate the change in language on page 6 about the GID-complex and Hh signaling components. Finally, I feel the authors have satisfactorily address the concerns Reviewer 1 raised.

Comments for the author

I feel the revised manuscript is satisfactory in its current form.

© 2022. Published by The Company of Biologists under the terms of the Creative Commons Attribution License (https://creativecommons.org/licenses/by/4.0/).
